# Transition-Metal-Free Click Polymerization Toward Poly(vinyl sulfide)s Endowed with AIE-Driven Noble Metal Sensing

**DOI:** 10.3390/polym18101202

**Published:** 2026-05-14

**Authors:** Liangcong Fan, Peisen Xu, Hongyu Wang, Zhifeng Cai, Juan Zuo, Cong Liu, Xiaohang Tan, Fengxiong Long, Hao Luo, Qingqing Gao

**Affiliations:** School of Materials Science and Engineering, Xiamen University of Technology, Xiamen 361024, China; fanliangcong@gmail.com (L.F.); 15939446407@139.com (P.X.); whongyu605@gmail.com (H.W.); czf3430324293@163.com (Z.C.); zuojuan@xmut.edu.cn (J.Z.); cuigu808@126.com (C.L.); 15959445032@163.com (X.T.); 15318720649@163.com (F.L.)

**Keywords:** alkyne–thiol click polymerization, poly(vinyl sulfide)s, aggregation-induced emission, fluorescent polymers, chemosensor

## Abstract

A novel transition-metal-free alkyne–thiol click polymerization with 100% atom economy is reported. Using *^t^*BuOLi as a catalyst at 80 °C, the polymerization efficiently yields poly(vinyl sulfide)s (PVSs) with molecular weights up to 11,800 g/mol and yields up to 91%. These sulfur-rich polymers exhibit high thermal stability (*T*_d_ up to 293 °C) and high refractive indices (1.8375–1.6383) across the visible range. By integrating abundant sulfur coordination sites with aggregation-induced emission (AIE) properties, the PVS aggregates serve as high-performance fluorescent chemosensors. The sensor enables exclusive, sensitive trace detection of Pd^2+^ and Au^3+^ with remarkable anti-interference capability and pH robustness (pH 1–7). Notably, an ultrafast response (1–2 min) for Pd^2+^ is achieved, with limits of detection (LOD) reaching 7.11 × 10^−7^ M for Pd^2+^ and 1.06 × 10^−6^ M for Au^3+^, and corresponding limits of quantification (LOQ) reaching 2.37 × 10^−6^ M and 3.53 × 10^−6^ M, respectively. This methodology offers a sustainable route to heteroatom-rich macromolecules for next-generation optical engineering and environmental monitoring.

## 1. Introduction

The accurate detection of noble metal ions, particularly palladium (Pd^2+^) and gold (Au^3+^), has emerged as a critical imperative driven by their economic value, environmental impact, and severe biological toxicity [1,2,3]. Due to their extreme scarcity in the Earth’s crust and high commercial value, monitoring these ions in industrial effluents is of tremendous economic significance for resource recovery and sustainable development. On the other hand, the widespread application of these metals inevitably leads to the discharge of Pd^2+^ and Au^3+^ into the environment. As non-biodegradable heavy metal pollutants, they persist in soil and aquatic systems and bioaccumulate through the food web, severely disrupting the ecological balance [4,5,6]. More alarmingly, from a toxicological perspective, Pd^2+^ and Au^3+^ act as typical soft acids and exhibit a profound binding affinity toward soft base sites in biological systems, such as sulfur-containing proteins, enzymes, and DNA. The intracellular accumulation of these highly reactive ions can irreversibly deactivate crucial enzymes and perturb normal cellular metabolism [7,8,9]. For instance, Au^3+^ is highly cytotoxic and can cause severe damage to the liver, kidneys, and peripheral nervous system, while continuous exposure to Pd^2+^ is known to trigger severe allergic reactions, asthma, and potential DNA damage [10,11,12]. Therefore, developing highly sensitive and selective chemosensors for the trace detection of Pd^2+^ and Au^3+^ is of paramount importance for environmental protection, human health monitoring, and precious metal recycling.

Among various analytical techniques, the luminescent detection of metal ions has garnered significant attention due to its exceptional sensitivity, selectivity, high signal-to-noise ratio, cost-effectiveness, and operational simplicity. Furthermore, molecular engineering allows for the precise tunability of probe molecules to target a wide array of analytes [13,14,15]. In these systems, synthetic receptors are vital for the selective capture of specific metal ions. These receptor units typically feature electron-rich donor atoms (e.g., N, O, P, and S) that act as Lewis bases [16,17,18,19]. Coordination between these basic receptors and target metal ions (Lewis acids) induces distinct photophysical changes in the adjacent luminescent core, enabling reliable detection. However, classic organic dyes, such as coumarin, rhodamine, fluorescein, and cyanine, as well as conventional fluorescent polymers, are inherently limited by their photophysical behavior. While they emit efficiently in dilute, mono-dispersed states, they frequently suffer from aggregation-caused quenching (ACQ) at higher concentrations or in the solid state due to detrimental π–π stacking interactions [20]. Because real-world sensing applications often require luminescent materials to operate as thin films or solid-state devices, this ACQ effect represents a significant bottleneck [21,22,23].

To circumvent this limitation, aggregation-induced emission (AIE) luminogens, such as the propeller-shaped tetraphenylethylene (TPE), have been widely leveraged. Unlike ACQ materials, AIEgens exhibit intense fluorescence in the aggregated state. Fundamentally, this phenomenon is driven by the restriction of intramolecular motions (RIM); specifically, physical constraints within the aggregates block the non-radiative rotation of TPE’s peripheral phenyl rings, thereby activating strong emission [21,24,25,26]. Consequently, integrating metal-specific receptors with AIE-active scaffolds represents a highly promising strategy for developing robust, solid-state metal ion sensors. To construct such AIE-active, metal-specific sensors, sulfur-rich macromolecules such as poly(vinyl sulfide)s (PVSs) are highly desirable. According to Pearson’s hard–soft acid–base (HSAB) theory, the abundant sulfur heteroatoms along the PVS backbone act as ideal soft bases to selectively capture soft acid noble metal ions [27,28,29]. While alkyne–thiol click polymerization represents a powerful route to access PVSs, conventional methodologies heavily rely on transition-metal catalysts [30]. The inevitable retention of trace heavy-metal residues within the resulting polymer matrix is highly detrimental, as it severely interferes with luminescent sensing by causing background quenching or false-positive signals. Consequently, developing a highly efficient, transition-metal-free click polymerization strategy for PVSs remains a significant challenge.

**Scheme 1:**
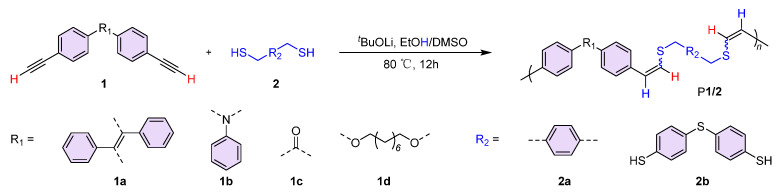
Alkoxide base-catalyzed nucleophilic polyaddition of aryldiynes and benzyl dithiols.

Herein, inspired by pioneering work on base-catalyzed small-molecule click reactions [31], a highly efficient, transition-metal-free alkyne–thiol click polymerization was successfully established, boasting a theoretical 100% atom economy. Under the catalysis of *t*-BuOLi at 80 °C for 12 h, the click polymerization proceeded robustly to generate a diverse series of poly(vinyl sulfide)s (Scheme 1). This versatile methodology delivers the target macromolecules in excellent yields (up to 91%) with remarkably high molecular weights (up to 11,800 g/mol). Benefiting from their unique structural features, the resulting polymers exhibit outstanding thermal stability and exceptional light refractivity across the visible wavelength range. More importantly, by leveraging their AIE characteristics and abundant sulfur coordination sites, the aggregated PVSs were successfully deployed as highly reliable fluorescent chemosensors. The sensor demonstrated extraordinary anti-interference capabilities and environmental robustness for the exclusive trace detection of Pd^2+^ and Au^3+^ via a drastic fluorescence quenching response.

## 2. Materials and Methods

### 2.1. Materials

**Scheme 2:**
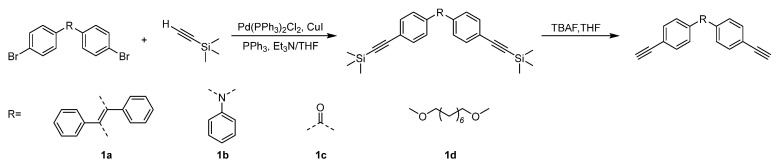
Synthetic route to monomers **1a**–**1d**.

1,4-benzenedimethanethiol (**2a**), 4,4′-thiodibenzenethiol (**2b**), and the catalyst lithium tert-butoxide (*^t^*BuOLi) were purchased from Aladdin. Monomers **1a**–**1d** were readily synthesized according to previously reported procedures [32,33,34] (Scheme 2) and purified by silica gel column chromatography. The starting materials for the synthesis of monomers **1a**–**1d**, including 1,2-bis(4-bromophenyl)-1,2-diphenylethene, 4,4′-dibromotriphenylamine, 4,4′-dibromobenzophenone, trimethylsilylacetylene, 1,8-bis(4-(bromoethynyl)phenoxy)octane, tetrabutylammonium fluoride (TBAF), and phenylacetylene, were also purchased from Aladdin. Solvents including dichloromethane (DCM), 1,2-dichloroethane (DCE), ethanol (EtOH), *N*,*N*-dimethylformamide (DMF), and dimethyl sulfoxide (DMSO) were purchased from J&K Scientific Ltd., Beijing, China. and dried over molecular sieves prior to use. Tetrahydrofuran (THF), hexane, other chemicals, and all metal salts (AgClO_4_, HAuCl_4_, CuCl_2_, CdCl_2_, Pd(NO_3_)_2_, PtCl_4_, MnCl_2_, NiCl_2_, Co(NO_3_)_2_, ZnCl_2_, KCl, Fe(NO_3_)_3_, CaCl_2_, Al(NO_3_)_3_, LiNO_3_, HgCl_2_, NaNO_3_, and MgCl_2_) were purchased from Aladdin Bio-Chem Technology Co., Ltd., Shanghai, China, Innochem Technology Co., Ltd., Beijing, China or Sinopharm Chemical Reagent Co., Ltd., Shanghai, China. Unless otherwise specified, all commercially available reagents and chemicals were used as received without further purification.

### 2.2. Monomer Synthesis

Monomers **1a**–**1d** were synthesized according to previously reported procedures (Scheme 2) [32,33,34], and their structures were sufficiently confirmed by NMR spectroscopy. Employing standard Schlenk techniques, the target compounds were conveniently prepared from trimethylsilylacetylene and their corresponding brominated precursors, namely 1,2-bis(4-bromophenyl)-1,2-diphenylethene, 4,4′-dibromotriphenylamine, 4,4′-dibromobenzophenone, and 1,8-bis(4-(bromoethynyl)phenoxy)octane.

The synthesis of **1a** is described below as a representative example. To a 250 mL three-necked round-bottom flask equipped with a magnetic stir bar were sequentially added 1,2-bis(4-bromophenyl)-1,2-diphenylethene (2.45 g, 5.0 mmol), Pd(PPh_3_)_2_Cl_2_ (140 mg, 0.2 mmol), CuI (76 mg, 0.40 mmol), and PPh_3_ (158 mg, 0.6 mmol). The flask was evacuated and backfilled with dry nitrogen three times. Subsequently, triethylamine (TEA, 80 mL) and THF (30 mL) were injected by syringe, followed by the addition of trimethylsilylacetylene (1.84 mL, 13 mmol). The reaction mixture was stirred at 50 °C overnight and monitored by TLC. Upon completion, the solvents were removed under reduced pressure. The residue was extracted with dichloromethane and water three times. The combined organic layers were concentrated and purified by silica gel column chromatography using DCM/hexane as the eluent to afford the TMS-protected intermediate as a white solid (2.49 g, 95% yield).

Subsequently, the obtained intermediate (2.10 g, 4.0 mmol) was dissolved in THF (50 mL) in a 250 mL single-necked round-bottom flask. A solution of TBAF (16 mL, 16.0 mmol) was then added dropwise, and the resulting mixture was stirred at room temperature for 12 h. After completion, the mixture was extracted with dichloromethane and deionized water. The organic phase was collected, concentrated, and purified by column chromatography using hexane/DCM (10:1, *v*/*v*) as the eluent to furnish monomer **1a** as a yellow solid.

Monomer **1a**: Yellow solid; yield: 84% (1.28 g). FT-IR (film), *ν* (cm^−1^): 3281 (≡C–H), 3064, 3027, 2109 (C≡C), 1595, 1491, 1440, 1255, 1106, 1071, 1020, 975, 916, 824, 761, 689, 663, 614. ^1^H NMR (500 MHz, CDCl_3_), *δ* (ppm): 7.29–7.22 (dd, *J* = 11.4, 8.2 Hz, 4H), 7.18–7.08 (dq, *J* = 9.3, 2.7 Hz, 6H), 7.05–6.96 (ddt, *J* = 13.4, 8.2, 4.6 Hz, 8H), 3.09–3.04 (d, *J* = 8.6 Hz, 2H). ^13^C NMR (125 MHz, CDCl_3_), *δ* (ppm): 144.27, 143.05, 141.06, 131.72, 131.52–131.24, 128.10, 127.94, 127.01, 120.29, 83.83.

Monomer **1b**: White solid; yield: 88% (460 mg). FT-IR (film), *ν* (cm^−1^): 3349 (≡C–H), 3021, 2919, 2097 (C≡C), 1654, 1581, 1483, 1417, 1312 (C–N), 1273, 1046, 1008, 951, 818, 755, 694, 638. ^1^H NMR (500 MHz, CDCl_3_), *δ* (ppm): 7.42–7.34 (m, 4H), 7.34–7.27 (m, 2H), 7.15–7.08 (m, 3H), 7.03–6.96 (m, 4H), 3.06 (s, 1H). ^13^C NMR (125 MHz, CDCl_3_), *δ* (ppm): 147.97, 146.83, 146.43, 133.32, 132.53, 129.71, 126.17, 125.27, 124.21, 122.60, 116.05, 115.62, 83.82, 76.64.

Monomer **1c**: White solid; yield: 82% (0.34 g). FT-IR (film), ν (cm^−1^): 3355 (≡C–H), 3023, 2917, 2080 (C≡C), 1640 (C=O), 1585, 1546, 1507, 1405, 1309, 1277, 1175, 1142, 1016, 926, 849, 779, 767, 673. ^1^H NMR (500 MHz, CDCl_3_), *δ* (ppm): 7.76–7.71 (dd, *J* = 8.3, 1.7 Hz, 4H), 7.62–7.56 (dd, *J* = 8.2, 1.7 Hz, 4H), 3.28–3.32 (s, 2H). ^13^C NMR (125 MHz, CDCl_3_), *δ* (ppm): 195.07, 137.15, 132.19, 129.97, 126.62, 82.84, 80.47.

Monomer **1d**: Brown solid; yield: 82% (0.46 g). FT-IR (film), *ν* (cm^−1^): 3292 (≡C–H), 3037, 2941, 2908, 2870, 2103 (C≡C), 1605, 1508, 1473, 1289, 1250, 1207, 1168, 1109, 1026, 987, 838, 818, 767, 728, 707, 669, 650. ^1^H NMR (500 MHz, CDCl_3_), *δ* (ppm): 7.46–7.36 (m, 4H), 6.87–6.78 (m, 4H), 3.95 (t, *J* = 6.5 Hz, 4H), 2.99 (s, 2H), 1.78 (p, *J* = 6.7 Hz, 4H), 1.50–1.36 (m, 8H). ^13^C NMR (125 MHz, CDCl_3_), *δ* (ppm): 159.64, 133.70, 114.58, 114.02, 83.89, 75.81, 68.12, 29.40, 29.26, 26.07.

### 2.3. Polymer Synthesis

Unless otherwise stated, all polymerization reactions were carried out in thick-walled glass tubes in air. The synthesis of P**1a**/**2a** is described below as a representative example. To a 15 mL thick-walled tube were sequentially added monomer **1a** (76 mg, 0.20 mmol), 1,4-benzenedimethanethiol (**2a**, 34 mg, 0.20 mmol), and *^t^*BuOLi (8 mg, 0.20 mmol). Subsequently, a solvent mixture of EtOH/DMSO (2.0 mL, 1:1, *v*/*v*) was introduced. The reaction mixture was then stirred at 80 °C for 12 h. Upon completion, the mixture was cooled to room temperature, diluted with dichloromethane (5 mL), and filtered through a short pad of silica gel directly into vigorously stirred hexane (50 mL) to precipitate the polymer. The resulting precipitates were collected by centrifugation and dried in a vacuum oven at 60 °C to a constant weight, affording the target polymer P**1a**/**2a**.

P**1a**/**2a**: Yellow powder; 86% yield. *M*_w_ = 11,800 g/mol; *M*_w_/*M*_n_ = 1.87. FT-IR (film), *ν* (cm^−1^): 3021, 2958, 2921, 2856, 1667, 1597, 1505, 1440, 1406, 1316, 1240, 1179, 1108, 1016, 953, 910, 838, 814, 763, 698, 626, 602, 573. ^1^H NMR (500 MHz, CDCl_3_), *δ* (ppm): 7.29 (d, *J* = 10.3 Hz), 7.20 (t, *J* = 8.6 Hz), 7.08 (d, *J* = 7.4 Hz), 7.05–6.82 (m), 6.66–6.35 (m, 1H), 6.35–6.06 (m, 3H), 4.05–3.82 (m, 4H). ^13^C NMR (125 MHz, CDCl_3_), *δ* (ppm): 143.81, 142.30, 140.78, 136.70, 135.03, 132.09–130.81, 129.33, 128.56–127.50, 126.92–126.50, 126.50–125.36, 125.06, 39.34.

P**1b**/**2a**: Light yellow powder; 45.7% yield. FT-IR (film), *ν* (cm^−1^): 3023, 2917, 1658 (C=C, vinylene), 1591, 1581, 1505, 1483, 1418, 1312, 1273, 1191, 1175, 1104, 1071, 993, 957, 940, 908, 818, 755, 694, 640, 620. ^1^H NMR (500 MHz, CDCl_3_), *δ* (ppm): 7.31 (d, *J* = 11.5 Hz), 7.28–7.15 (m), 7.15–6.78 (m), 6.40–6.02 (m, 2H), 4.80 (s, 4H).

P**1c**/**2a**: Orange-yellow powder; 37.5% yield. FT-IR (film), *ν* (cm^−1^): 2923, 2859, 1646 (C=O), 1595, 1505, 1405, 1273, 1167, 1102, 1020, 930, 846, 634.

P**1d**/**2a**: Yellow powder; 74.4% yield. FT-IR (film), *ν* (cm^−1^): 3027, 2925, 2854, 1663, 1599, 1505, 1469, 1419, 1301, 1244, 1169, 1109, 1016, 910, 828, 700, 641, 616.

P**1a**/**2b**: Brown powder; 76.7% yield. FT-IR (film), *ν* (cm^−1^): 3019, 1652 (C=C), 1583, 1473, 1395, 1312, 1187, 1089, 1014, 951, 812, 755, 696, 634, 612.

### 2.4. Model Compound Synthesis

**Scheme 3:**
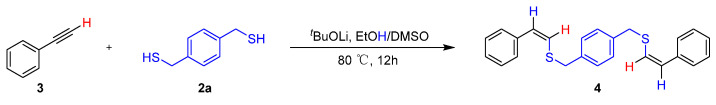
Synthetic route to Model Compound **4**.

The model compound was synthesized via the reaction of 1,4-benzenedimethanethiol (**2a**) with phenylacetylene (Scheme 3). To a 50 mL single-necked round-bottom flask were sequentially added **2a** (340 mg, 2.0 mmol) and *^t^*BuOLi (160 mg, 2.0 mmol). A solvent mixture of EtOH and DMSO (2.0 mL each) was then introduced to completely dissolve the solids, followed by the dropwise addition of phenylacetylene (0.438 mL, 4.0 mmol). The reaction mixture was heated and stirred at 80 °C for 12 h. Upon completion, the mixture was cooled to room temperature, and the solvents were removed under reduced pressure. The residue was diluted with deionized water and extracted with DCM. The combined organic layers were concentrated and purified by silica gel column chromatography using DCM/hexane as the eluent to afford the model compound **4** as white powder (643 mg, 86% yield). FT-IR (film), *ν* (cm^−1^): 3046, 2919, 1591, 1509, 1489, 1440, 1356, 1201, 1154, 1075, 1026, 932, 906, 887, 838, 810, 779, 677, 652, 612. ^1^H NMR (500 MHz, CDCl_3_), *δ* (ppm): 7.48–7.42 (m, 4H), 7.34 (s, 8H), 7.24–7.17 (m, 2H), 6.45 (d, *J* = 10.9 Hz, 2H), 6.23 (d, *J* = 10.9 Hz, 2H), 3.98 (s, 4H). ^13^C NMR (125 MHz, CDCl_3_), *δ* (ppm): 136.95, 136.76, 129.45, 128.81, 128.37, 126.90, 126.09, 39.32. HRMS (ESI): *m*/*z* [M + H]^+^ calcd for C_24_H_23_S_2_^+^: 375.1236, found, 375.1236.

### 2.5. Fabrication of Polymer Films

Polymer thin films were fabricated by spin-coating. Briefly, the polymer was dissolved in 1,2-dichloroethane (40 mg mL^−1^) and passed through a syringe filter. The resulting clear filtrate was spin-coated onto silicon wafers using a KW-4A spin coater at 400 rpm for 6 s, followed by 1000 rpm for 1 min. Subsequently, the wet films were dried in an oven at 60 °C for 30 min to remove residual solvent.

### 2.6. Metal Ions Detection

#### 2.6.1. Fluorescence Selectivity Studies

Aqueous stock solutions (1.0 mM) of various metal ions were prepared by dissolving their corresponding salts (AgClO_4_, HAuCl_4_, CuCl_2_, CdCl_2_, Pd(NO_3_)_2_, PtCl_4_, MnCl_2_, NiCl_2_, Co(NO_3_)_2_, ZnCl_2_, KCl, Fe(NO_3_)_3_, CaCl_2_, Al(NO_3_)_3_, LiNO_3_, HgCl_2_, NaNO_3_ and MgCl_2_) in deionized water. A stock solution of the polymer P**1a**/**2a** (1.0 mM) was prepared in THF. For the selectivity assays, 1.0 mL of the respective metal ion stock solution was diluted with 7.0 mL of deionized water, followed by the addition of 400 μL of the P**1a**/**2a** stock solution and 1.6 mL of THF. A blank control group was prepared under identical conditions by substituting the metal ion solution with 1.0 mL of deionized water. The final testing mixtures (total volume: 10.0 mL) contained 100 μM of the metal ion and 40 μM of P**1a**/**2a**. Once thoroughly mixed, the fluorescence spectra of the resulting solutions were recorded immediately using a fluorescence spectrophotometer (F-7000, Hitachi High-Technologies Corporation, Tokyo, Japan).

#### 2.6.2. Anti-Interference Assay

To evaluate the competitive recognition capabilities of the polymer, an ionic immunity test was conducted. Briefly, 1.0 mL of the target ion stock solution (Pd^2+^ or Au^3+^) and 1.0 mL of an interfering metal ion stock solution were added to 6.0 mL of deionized water. This aqueous mixture was subsequently combined with 400 μL of the P**1a/2a** stock solution and 1.6 mL of THF. The final testing system maintained a concentration of 100 μM for each metal ion and 40 μM for the polymer. Fluorescence measurements were performed immediately after homogenization.

#### 2.6.3. Fluorescence Titration and Sensitivity Assays

To determine the detection sensitivity, aliquots of the Pd^2+^ stock solution (0–200 μL) were diluted with corresponding volumes of deionized water (8.0–7.8 mL) to maintain a constant aqueous volume of 8.0 mL. These solutions were then thoroughly mixed with 400 μL of the P**1a**/**2a** stock solution and 1.6 mL of THF. This procedure generated a series of testing solutions with varying Pd^2+^ concentrations (0–20 μM) but a constant P**1a**/**2a** concentration of 40 μM. A completely analogous titration protocol was employed to investigate the sensitivity toward Au^3+^ ions, utilizing a final Au^3+^ concentration gradient of 0–36 μM.

#### 2.6.4. Evaluation of Environmental Robustness via pH Titrations

Aqueous solutions with varying pH values were initially prepared by adjusting deionized water with dilute HCl or NaOH. To evaluate the pH stability of the pristine polymer, a blank solution of P**1a**/**2a** was prepared by mixing 0.4 mL of the polymer stock solution and 1.6 mL of THF into 8.0 mL of the pH-adjusted water. For the sensing assays at different pH levels, 1.0 mL of the respective noble metal ion stock (Pd^2+^ or Au^3+^) was first diluted in 7.0 mL of the pH-adjusted water. Subsequently, 0.4 mL of the P**1a**/**2a** stock and 1.6 mL of THF were added to this aqueous mixture. All resulting solutions were thoroughly homogenized prior to measurement, maintaining uniform final concentrations of 40 μM for the polymer P**1a**/**2a** and 100 μM for the target metal ions.

### 2.7. Characterization

#### 2.7.1. Nuclear Magnetic Resonance (NMR) Spectroscopy

All ^1^H and ^13^C nuclear magnetic resonance spectra were recorded on a Bruker AVANCE III 500 spectrometer operating at frequencies of 500 MHz for ^1^H and 125 MHz for ^13^C, respectively. Measurements were performed at 298 K using deuterated chloroform (CDCl_3_) as the solvent. The sample concentrations were prepared at approximately 5 mg mL^−1^ for ^1^H NMR and 15–20 mg mL^−1^ for ^13^C NMR characterizations. Chemical shifts (*δ*) are reported in parts per million (ppm) referenced to tetramethylsilane (TMS, *δ* = 0.00 ppm) as an internal standard or the residual solvent signals (*δ* = 7.26 ppm for ^1^H NMR and *δ* = 77.16 ppm for ^13^C NMR).

#### 2.7.2. Fourier Transform Infrared (FT-IR) Spectroscopy

FT-IR spectra were collected on a spectrometer equipped with an attenuated total reflection (ATR) accessory (ATR, Bruker Corporation, Karlsruhe, Germany). Solid powder samples were placed directly onto the ATR crystal and measured under ambient air at room temperature. The spectra were recorded over a wavenumber range of 4000–400 cm^−1^ with a resolution of 0.8 cm^−1^.

#### 2.7.3. High-Resolution Mass Spectrometry (HRMS)

High-resolution mass spectrometry (HRMS) was performed on a quadrupole time-of-flight (Q-TOF) mass spectrometer (Q-TOF, Agilent Technologies, Inc., Santa Clara, CA, USA) equipped with an electrospray ionization (ESI) source, operating in positive ion acquisition mode. The operating conditions utilized a mobile phase consisting of pure water and methanol (*v*/*v*: 1:3) at a flow rate of 10 μL/min; the sheath gas temperature was set to 350 °C, the nebulizer pressure to 35 psi, and the drying gas (nitrogen) to a temperature of 300 °C.

#### 2.7.4. Gel Permeation Chromatography (GPC)

The molecular weights and polydispersity indices of the polymers were evaluated using a e2695 GPC system equipped with a refractive index (RI) detector (GPC, Waters Corporation, Milford, MA, USA). Measurements were performed using THF as the eluent at a flow rate of 1.0 mL min^−1^ at 35 °C. An injection volume of 10 μL was used, and the system was calibrated against monodisperse polystyrene (PS) standards.

#### 2.7.5. Thermal Analysis (TGA and DSC)

Thermogravimetric analysis (TGA) and differential scanning calorimetry (DSC) were simultaneously performed on a 449 F5 thermal analyzer (STA, Netzsch Gerätebau GmbH, Selb, Germany). Approximately 5–10 mg of each sample was accurately weighed into an alumina crucible. To eliminate the thermal history and remove any residual solvent, the sample was first heated from room temperature (RT) to 150 °C, cooled back to RT, and subsequently heated to 800 °C. All measurements were conducted under a continuous argon atmosphere at a heating/cooling rate of 10 °C min^−1^.

#### 2.7.6. UV-Vis Absorption Spectroscopy

Ultraviolet–visible (UV-vis) absorption spectra of the polymers were recorded on a UV-2700 spectrophotometer (UV, Shimadzu Corporation, Kyoto, Japan) using standard 10 mm path-length quartz cuvettes. The polymer samples were dissolved in THF at a uniform concentration of 40 μM. Spectra were collected at room temperature over a wavelength range of 200–800 nm.

#### 2.7.7. Photoluminescence (PL) Spectroscopy

Photoluminescence (PL) measurements were performed on a fluorescence spectrophotometer (F-7000, Hitachi High-Technologies Corporation, Tokyo, Japan). A polymer stock solution (1.0 mM) was prepared by dissolving an accurately weighed amount of the sample in 10 mL of THF. This stock was then diluted 25-fold to obtain a final testing solution with a concentration of 40 μM. The PL emission spectra were recorded using a 10 mm quartz cuvette over a wavelength range of 370–650 nm at an excitation wavelength of 350 nm.

#### 2.7.8. Spectroscopic Ellipsometry (Refractive Index)

The refractive indices (*n*) of the spin-coated polymer thin films (on silicon wafers) were measured using a SENTECH SE800 DUV spectroscopic ellipsometer (SE800, SENTECH Instruments GmbH, Berlin, Germany). The instrument features a tunable wavelength from 190 to 950 nm, and the specific measurements for the films were carried out over a wavelength range of 400–800 nm at room temperature.

## 3. Results

### 3.1. Monomer Synthesis and Discussion

Monomers **1a**–**1d** were prepared through a robust two-step synthetic protocol consisting of a standard Sonogashira cross-coupling followed by the cleavage of trimethylsilyl (TMS) protecting groups, as illustrated in Scheme 2. The initial palladium-catalyzed coupling between various aryl bromides and trimethylsilylacetylene was conducted under mild conditions, delivering the TMS-protected intermediates in high yields (up to 95%). This reaction demonstrates excellent functional group tolerance, effectively accommodating diverse molecular architectures including tetraphenylethylene (**1a**), triphenylamine (**1b**), benzophenone (**1c**), and aliphatic-linked phenyl units (**1d**). A key advantage of this two-step strategy is the use of TMS-protected acetylene, which significantly minimizes undesired Glaser-type homocoupling side reactions, thereby guaranteeing high selectivity toward the target aryldiyne monomers. The exact chemical structures of monomers **1a**–**1d** were thoroughly verified by ^1^H and ^13^C NMR spectroscopy (Figures S8–S15). In the ^1^H NMR spectra, the characteristic resonances of the substituted phenyl rings emerge in the aromatic region of *δ* 6.8–7.7 ppm. Crucially, the diagnostic terminal alkynyl proton (≡C–H) resonates as a sharp singlet at *δ* 3.0–3.3 ppm, unambiguously confirming the complete deprotection and the presence of the reactive diyne functionality. These assignments are firmly supported by the ^13^C NMR spectra, which display the distinctive sp-hybridized acetylenic carbons at *δ* 80–90 ppm and the aromatic backbone carbons spanning *δ* 120–150 ppm. Moreover, monomer-specific functionalities are clearly resolved, such as the carbonyl carbon of **1c** (*δ* 195 ppm) and the aliphatic/ether carbons of **1d** (*δ* 20–70 ppm). Overall, these spectroscopic profiles perfectly match their theoretical structures and the reported literature values, thereby confirming the successful synthesis of highly pure monomers prerequisite for the subsequent click polymerization.

### 3.2. Polymerization

To develop this two-component click polymerization of terminal diynes and benzyl dithiols as a facile strategy towards PVSs, we optimized the polymerization conditions using **1a** and **2a** as model monomers. The effects of several reaction parameters including solvent, solvent volume ratios, polymerization temperatures and time course on the polymerization results were carefully examined (Table 1).

The choice of solvent is crucial for both the efficiency of the polymerization and the solubility of the growing polymer chains. Although pure EtOH was identified as the optimal solvent for the small-molecule model reaction [31], its exclusive use in the polymerization resulted in premature precipitation of the polymer chains due to their poor solubility, severely limiting the reaction. Therefore, various solvent mixtures were evaluated for the polymerization of **1a** and **2a** at 60 °C under an ambient atmosphere for 12 h in the presence of *^t^*BuOLi (Table 1, entries 1–5). The H_2_O/DMSO and EtOH/DCE co-solvent systems yielded only trace amounts of polymeric products. In contrast, mixing EtOH with DMSO, DMF, or DCM afforded P**1a**/**2a** with moderate weight-average molecular weights (*M*_w_ = 8600, 6700, and 7900 g/mol, respectively). Among these, the EtOH/DMSO mixture afforded the highest yield of 71% and was thus identified as the optimal solvent system. Subsequent investigation of the co-solvent ratio revealed that an equal volume mixture (1:1, *v*/*v*) of EtOH and DMSO delivered the best results. This synergistic effect occurs because EtOH acts as a vital proton donor for the reaction, while DMSO ensures adequate solvation of the propagating polymer chains to mitigate premature precipitation. Deviating from this optimal ratio led to drastically reduced yields and the formation of only short oligomers (Table 1, entries 6–9).

Next, the effect of temperature was investigated in the EtOH/DMSO (1:1, *v*/*v*) system (Table 1, entries 10–12). Elevating the temperature from 50 to 80 °C led to a concurrent increase in both molecular weight and yield, culminating in an *M*_w_ of 11,800 g/mol and a yield of 91% at 80 °C. This improvement is attributed to the accelerated reaction kinetics and the reduced solution viscosity at elevated temperatures, which jointly enhance the mobility and reactivity of the macromolecular polymer chains. Finally, the optimization of reaction time at 80 °C (Table 1, entries 13–15) demonstrated that both molecular weight and yield increased over time, reaching a maximum at 12 h. Consequently, the optimal polymerization conditions were established, tBuOLi as the catalyst, EtOH/DMSO (1:1, *v*/*v*) as the solvent, and heating at 80 °C for 12 h under an ambient atmosphere. Importantly, it should be noted that this systematic optimization was exclusively monitored using the **1a**/**2a** monomer pair. Polymers derived from the other rigidly structured diyne monomers exhibit highly inflexible backbones. This inherent structural rigidity, compounded by strong interchain interactions—including stacking and dispersion forces driven by the incorporated sulfur atoms [35,36]—triggers severe premature precipitation during the polymerization process. Consequently, their profound insolubility in common organic solvents precludes continuous molecular weight monitoring via GPC.

### 3.3. Structural Characterization

**Figure 1:**
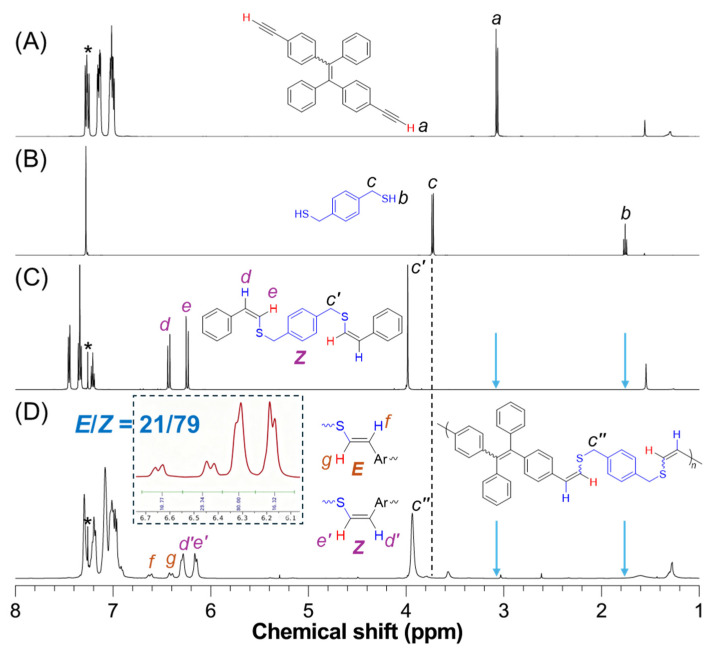
^1^H NMR spectra of (**A**) **1a**, (**B**) **2a**, (**C**) model compound **4** and (**D**) P**1a**/**2a** in CDCl_3_. The solvent peaks were marked with asterisks.

**Figure 2:**
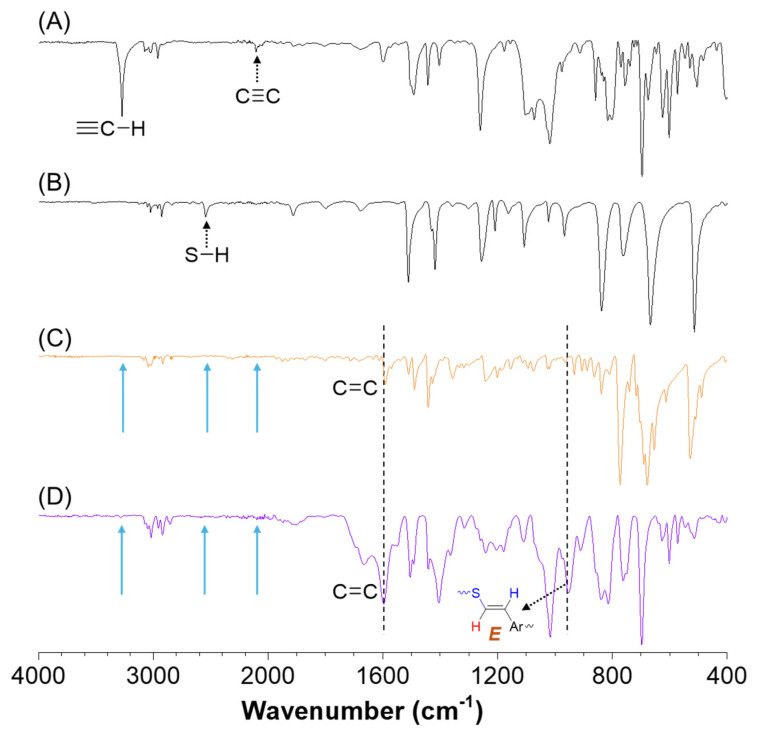
IR spectra of (**A**) **1a**, (**B**) **2a**, (**C**) model compound **4** and (**D**) P**1a**/**2a**.

To verify the successful execution of the click polymerization and provide a precise structural benchmark for the resulting macromolecules, a model reaction between phenylacetylene **3** and dithiol **2a** was conducted under similar conditions (Scheme 3). This reaction selectively produced the *Z*-isomer model compound **4**, whose structure was unambiguously confirmed by ^1^H NMR, ^13^C NMR, and high-resolution mass spectrometry (ESI-HRMS) (Figure 1, Figure 2 and Figure S4). Due to its high crystallinity and well-defined molecular weight, compound **4** offers a reliable “standard” for the vinyl sulfide linkage. Specifically, the sharp chemical shifts of the vinylic protons (*δ* 6.45 and 6.23 ppm) and the methylene protons (*δ* 4.0 ppm) in compound **4** serve as critical diagnostic references. By extrapolating these clear spectroscopic observations to the broader and more complex resonance signals of the polymers, the chemical integrity and the *Z*-isomer-dominant stereochemistry of the poly(vinyl sulfide) backbone were firmly confirmed. Additionally, the structures of all synthesized polymers were further corroborated by FT-IR spectroscopy (Figure S2).

To elucidate the polymer structure, the ^1^H NMR spectra of monomers **1a** and **2a**, model compound **4**, and polymer P**1a**/**2a** are systematically compared in Figure 1. In the spectra of the monomers, the terminal alkynyl protons of **1a** appear at *δ* 3.0 (peak a), while the thiol and adjacent methylene protons of **2a** resonate at *δ* 1.75 (peak b) and *δ* 3.70 (peak c), respectively. Following polymerization, the diagnostic peaks for both the alkyne and thiol protons completely disappear in the spectra of **4** and P**1a**/**2a**, indicating quantitative functional group consumption. Concurrently, the formation of new carbon–sulfur bonds induces a downfield shift of the methylene protons to *δ* 4.0 (peak c′) in model compound **4** and to *δ* 3.90 (peak c″) in polymer P**1a**/**2a**. Crucially, new resonances assigned to the vinylic protons emerge post-reaction. For the *Z*-configured model compound **4**, these vinylic protons appear at *δ* 6.45 and *δ* 6.23 (peaks d and e). In the spectrum of P**1a**/**2a**, the corresponding broad peaks for the *Z*-isomer are observed at *δ* 6.29 (peak d′) and *δ* 6.17 (peak e′). Additionally, two minor broad resonances emerge at *δ* 6.64 (peak f) and *δ* 6.42 (peak g) in the spectrum of P**1a**/**2a**, which belong to the vinylic protons of the *E*-isomer. Based on the integration of these vinylic proton signals, the *Z*/*E* stereoisomeric ratio of the polymer backbone is determined to be 79:21. Furthermore, the characteristic broadening of these resonance peaks in the P**1a**/**2a** spectrum verifies its macromolecular nature. Finally, ^13^C NMR spectroscopy (Figure S1) further corroborates these structural assignments, as the terminal alkyne carbon signal of **1a** at *δ* 83.66 is entirely absent in the spectra of both **4** and P**1a**/**2a**. Taken together, the NMR profiles of P**1a**/**2a** exhibit excellent agreement with those of the well-defined model compound **4**, unambiguously confirming the successful synthesis and precise chemical structure of the target polymers.

FT-IR spectroscopy was employed to further elucidate the chemical structures of the resulting polymers. As depicted in Figure 2, the FT-IR spectrum of monomer **1a** exhibits characteristic absorption bands at 3281 and 2109 cm^−1^, assigned to the ≡C–H and C≡C stretching vibrations of the terminal alkyne groups, respectively. For monomer **2a**, the diagnostic S–H stretching vibration is observed at 2544 cm^−1^. Following polymerization, these characteristic monomeric resonances completely vanish in the spectrum of P**1a**/**2a**, verifying the quantitative consumption of the ethynyl and mercapto groups during the polyaddition process. Concurrently, a new absorption band emerges at 1593 cm^−1^ in the spectra of both P**1a**/**2a** and model compound **4**, corresponding to the stretching vibration of the newly formed vinylic C=C backbone. Notably, a distinct peak at 954 cm^−1^, attributed to the out-of-plane bending vibration of the E-isomeric vinylic C–H bonds, is clearly visible in the spectrum of P**1a**/**2a**. The absence of this peak in the spectrum of model compound **4**, which exists exclusively as the *Z*-isomer, further corroborates the presence of a minor fraction of *E*-isomeric repeating units within the polymer backbone. These observations are in excellent agreement with the aforementioned NMR analysis, collectively confirming the successful alkyne–thiol click polymerization and the precise formation of the poly(vinyl sulfide) structure.

Furthermore, the successful syntheses of the broader P**1**/**2** polymer series were unambiguously confirmed via solid-state FT-IR spectroscopy (Figure S2). The spectra for all these resulting polymers display the complete disappearance of both the alkyne ≡C–H (~3290 cm^−1^) and thiol S–H (~2550 cm^−1^) stretching bands, accompanied by the emergence of the characteristic vinylic C=C absorption (~1600 cm^−1^). This robust spectral evidence firmly validates that the step-growth click polymerizations proceeded successfully, generating the target macromolecular structures despite the premature precipitation of the growing chains.

### 3.4. Reaction Mechanism

**Scheme 4:**
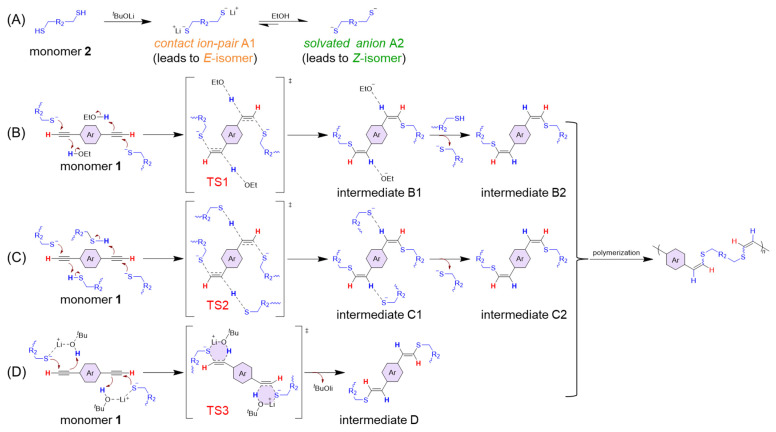
Proposed mechanism for the transition-metal-free alkyne–thiol click polymerization yielding poly(vinyl sulfide)s. (**A**) Deprotonation of dithiol monomer **2** by *^t^*BuOLi to generate the active thiolate species, which exists in an equilibrium between the contact ion-pair **A1** and the solvated anion **A2** in the presence of a protic solvent. (**B**) The *Z*-stereoselective pathway via transition state TS1, where EtOH serves as the proton donor during the anti-addition of the solvated anion onto diyne monomer **1**. (**C**) An alternative *Z*-stereoselective pathway via TS2, utilizing an unreacted thiol group as the proton donor. (**D**) The *E*-stereoselective pathway via TS3, driven by the contact ion-pair **A1** and the coordination of *^t^*BuOLi.

Based on the small-molecule model reactions, a detailed mechanism for this base-catalyzed alkyne–thiol polyaddition is proposed [31,37,38]. Initially, the base *^t^*BuOLi deprotonates the dithiol monomer, generating a highly nucleophilic thiolate anion that exists in a dynamic equilibrium between a contact ion-pair **A1** and a solvated anion **A2** (Scheme 4A). Subsequently, the thiolate undergoes a regioselective anti-Markovnikov nucleophilic attack on the terminal alkyne of the diyne monomer. As elucidated by the small-molecule model study [31], this addition does not proceed via a discrete, long-lived vinyl carbanion, but rather through concerted pathways. For the solvated anion **A2**, the attack is anti-periplanar and assisted by hydrogen bonding from a protic donor (EtOH or the thiol monomer), proceeding through concerted transition states TS1 or TS2 to stereospecifically afford the *Z*-isomer (Scheme 4B,C). Notably, the binary solvent system EtOH/DMSO plays an indispensable and dual role in this polymerization. As a strongly polar aprotic solvent, DMSO effectively solvates Li+, driving the dissociation of the contact ion pair **A1** into the solvated anion **A2**. By leaving the thiolate as a naked and highly reactive species, DMSO significantly accelerates the propagation rate. Furthermore, DMSO possesses an exceptional solvating capacity for the newly formed rigid aromatic polymer backbones, preventing their premature precipitation and keeping the reactive chain ends fully extended in the solution.

A fascinating stereochemical divergence was observed between the model compound and the final polymer. The small-molecule model reaction exclusively afforded the *Z*-isomer, whereas the polymer P**1a**/**2a** exhibited a mixed stereoregularity (*Z*/*E* = 79:21). This discrepancy can be perfectly rationalized by the shifting equilibrium between **A1** and **A2** within the evolving macromolecular microenvironment. In the unhindered small-molecule model reaction, the reactive species are uniformly dispersed in the mixed solvent. DMSO efficiently solvates Li^+^, shifting the equilibrium almost entirely toward the solvated anion **A2**. The abundant proton donor EtOH can effortlessly approach this fully exposed active site, driving the reaction exclusively through the concerted, *Z*-selective pathways (Scheme 4B,C). In sharp contrast, during the macromolecular polymerization, the microenvironment around the reactive center undergoes dramatic changes. As the polymer chain grows, the bulky and rigid polymer backbone creates severe steric hindrance. More importantly, the strong interaction between the polymer chain and DMSO forms a dense local solvation shell around the propagating chain ends. This sterically hindered and DMSO-rich microenvironment significantly restricts the free diffusion of EtOH molecules, causing a substantial fraction of the reactive centers to exist in the contact ion-pair **A1** state. The tight ion pairing in **A1** partakes in a divergent reaction pathway via a six-membered cyclic transition state TS3 coordinated by the lithium cation, which inherently leads to the thermodynamically more stable *E*-isomer (Scheme 4D). Thus, the dynamic competition between the **A2**-mediated concerted pathways (leading to *Z*) and the **A1**-mediated cyclic transition state pathway (leading to *E*) within the sterically restricted macromolecular microenvironment ultimately dictates the mixed *Z*/*E* stereoregularity in the resulting polymers.

### 3.5. Solubility, Thermal Stability, Light Refraction and Photophysical Properties

**Figure 3:**
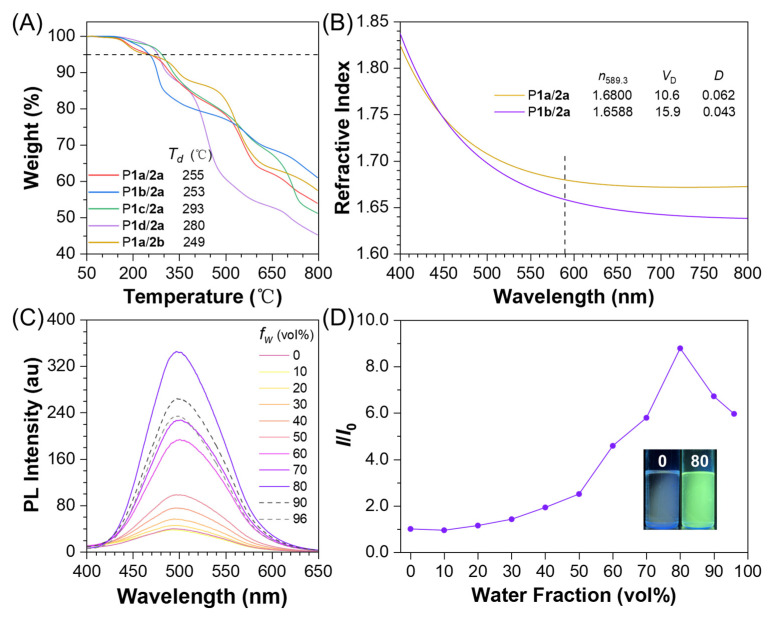
(**A**) TGA thermograms of P**1**/**2** recorded under argon at a heating rate of 10 °C/min. (**B**) Wavelength dependence of refractive index (*n*) of thin films of P**1a**/**2a** and P**1b**/**2a**. Abbreviation: *ν*_D_ = Abbé number = (*n*_D_ − 1)/(*n*_F_ − *n*_C_), where *n*_D_, *n*_F_, and *n*_C_ are the *n* values at wavelengths of 589.2, 486.1, and 656.3 nm, respectively, *D* = chromatic dispersion = 1/*ν*_D_. (**C**) PL spectra of P**1a**/**2a** in THF/H_2_O mixtures with different water fractions (*f*_w_, vol%). (**D**) Plot of the relative PL intensity (*I*/*I*_0_) at 500 nm versus the water fraction. *I*_0_ represents the PL intensity in pure THF. Inset picture: corresponding fluorescence photographs of P**1a**/**2a** in THF/H_2_O mixtures with *f*_w_ = 0 and 80% taken under a 365 nm hand-held UV lamp. Experimental conditions: [P**1a**/**2a**] = 40 μM, *λ*_ex_ = 350 nm.

The solubility of the resulting polymers was highly structure-dependent. Thanks to the bulky and twisted TPE units that disrupt dense chain packing, P**1a**/**2a** exhibited excellent macroscopic solubility, while P**1b**/**2a** showed limited but viable solubility. In sharp contrast, all other polymers precipitated as intractable solids due to the synergistic effect of rigid stacking and strong dispersion forces from the sulfur atoms [35]. The thermal properties of the PVSs were evaluated by TGA under argon. As depicted in Figure 3A, all polymers exhibit good thermal stability with decomposition temperatures *T*_d_ ranging from 249 °C to 293 °C. Additionally, owing to their highly conjugated and densely aromatic structural backbones, P**1a**/**2a**, P**1a**/**2b**, and P**1b**/**2a** exhibit remarkably high char yields of over 55%. Such exceptional thermal stability and rich carbon residue make these sulfur-containing polymers highly promising candidates for subsequent conversion into functional carbonaceous nanomaterials, paving the way for their specific applications in advanced catalytic fields [39,40]. Furthermore, DSC revealed no distinct glass transition temperatures *T*_g_ for any polymers prior to their thermal decomposition (Figure S5).

High-refractive-index polymers (HRIPs, *n* larger than 1.50) are highly desired for optoelectronic applications but often suffer from poor solubility and low transparency [41,42,43]. According to the Lorentz–Lorenz equation, introducing polarizable heteroatoms with high molar refractions is an effective strategy to enhance the refractive index (RI) [44,45]. Since P**1a**/**2a** and P**1b**/**2a** contain abundant rigid aromatic rings and highly polarizable sulfur atoms, they are structurally predisposed for good light refractivity. Despite their dense backbones, their sufficient solubility allowed the fabrication of uniform thin films via spin-coating. As shown in Figure 3B, these films demonstrated outstanding refractivity of 1.8375–1.6383 across the visible range 400–800 nm. Specifically, at 589.3 nm, the values for P**1a**/**2a** and P**1b**/**2a** reached 1.6800 and 1.6588, respectively, significantly outperforming conventional optical polymers (*n* = 1.49–1.58) [46]. Furthermore, based on the RI-wavelength dependence, the Abbé numbers *ν*_D_ for P**1a**/**2a** and P**1b**/**2a** were determined to be 10.6 and 15.9, with corresponding chromatic dispersions *D* of 0.062 and 0.043, respectively. Such low values highlight a classic optical trade-off, wherein the highly polarizable, extensively conjugated aromatic backbones significantly elevate the refractive index while concurrently inducing strong wavelength-dependent light dispersion. This combination of processability and high RI makes these sulfur-containing polymers promising candidates for next-generation photonic applications.

Then, photophysical properties of the synthesized PVSs were systematically investigated. The UV-vis absorption spectra of the synthesized polymers showed maximum peaks at 290–350 nm (Figure S6A). Regarding photoluminescence, P**1c**/**2a** and P**1d**/**2a** were found to be essentially non-emissive, which is fundamentally dictated by their specific monomeric structures. For P**1c**/**2a**, despite possessing a conjugated benzophenone moiety, the intrinsic *n*–*π*∗ transition associated with the carbonyl group facilitates rapid non-radiative decay pathways such as intersystem crossing, effectively quenching the fluorescence [47]. For P**1d**/**2a**, the incorporation of the aliphatic spacer completely disrupts the *π*–conjugation along the polymer backbone, eliminating any effectively conjugated fluorogenic core. In contrast, polymers endowed with classic fluorogenic cores exhibited distinct emission profiles in pure THF. P**1b**/**2a** displayed a strong emission band centered at ~445 nm. Meanwhile, the TPE-based polymers P**1a**/**2a** and P**1a**/**2b** underwent a significant bathochromic shift to ~495 nm, accompanied by substantially attenuated intensities (Figure S6B).

Afterwards, the PL behaviors of P**1a**/**2a** and P**1b**/**2a** were systematically investigated in THF/water mixtures. P**1b**/**2a** exhibited typical ACQ behavior, with its emission intensity continuously dropping as the water fraction increased (Figure S7). This is further corroborated by its exceptionally low absolute photoluminescence quantum yield (PLQY) of 0.3% in pure THF. In contrast, P**1a**/**2a** exhibited a pronounced AIE characteristic (Figure 3C,D). As *f*_w_ in the solvent mixture gradually increased, the PL intensity of the polymer grew continuously, reaching a maximum at an *f*_w_ of 80% with a remarkable 8.8-fold enhancement (*I*/*I*_0_ = 8.8). Correspondingly, the absolute PLQY of P**1a**/**2a** in the aggregated state reached up to 5.4%, standing in sharp contrast to the weakly emissive P**1b**/**2a**. Then, as *f*_w_ was further elevated to 90% and beyond, the PL intensity experienced a noticeable decrease, indicating P**1a**/**2a**’s AIE nature.

### 3.6. Metal Ion Detections

**Figure 4:**
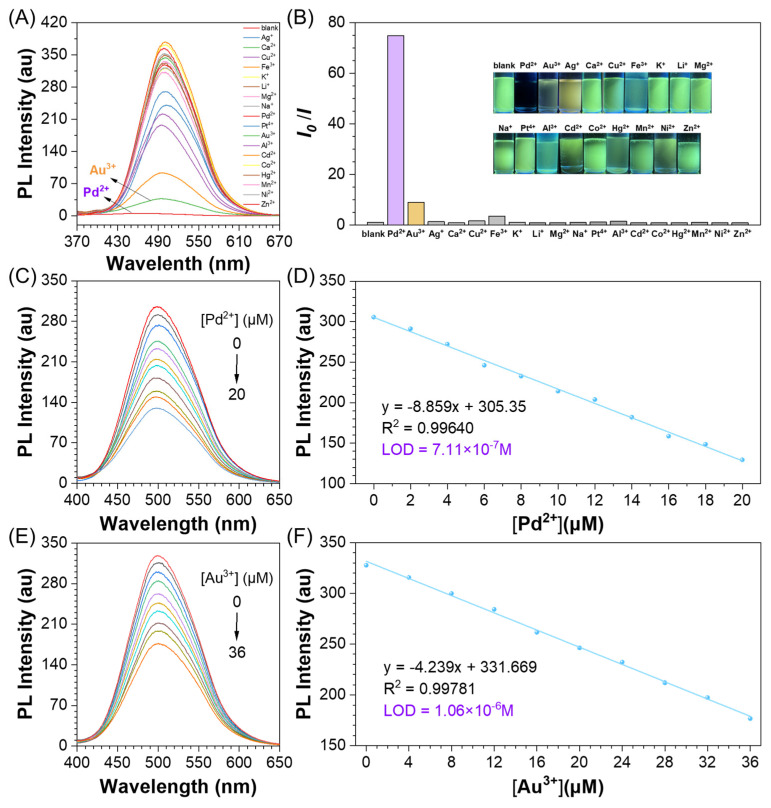
(**A**) PL spectra of P**1a**/**2a** upon the addition of various metal ions. (**B**) Relative PL intensity (*I*/*I*_0_) of P**1a**/**2a** at 500 nm in the presence of different metal ions. *I*_0_ and *I* represent the PL intensity in the absence and presence of metal ions, respectively. Inset picture: corresponding fluorescence photographs taken under a 365 nm hand-held UV lamp. PL spectra of P**1a**/**2a** upon the gradual addition of (**C**) Pd^2+^ (0–20 μM) and (**D**) Au^3+^ (0–36 μM). Corresponding linear fitting plots of the PL intensity at 500 nm versus the concentration of (**E**) Pd^2+^ and (**F**) Au^3+^ for the determination of the limit of detection (LOD). Experimental conditions: [P**1a**/**2a**] = 40 μM in THF/H_2_O mixture (*v*/*v* = 2:8), [metal ions] = 100 μM, *λ*_ex_ = 350 nm.

Herein, taking advantage of the AIE property of P**1a**/**2a** and the abundance of sulfur heteroatoms on its main chains that could coordinate with metal ions, its aggregates prepared in a THF/water mixture with a *f*_w_ of 80% were used to detect metal ions. To systematically evaluate the sensing performance of P**1a**/**2a**, its fluorescence response was initially screened against a comprehensive panel of 16 representative background metal ions, including common alkali, alkaline earth, and transition metal ions (Na^+^, K^+^, Li^+^, Mg^2+^, Ca^2+^, Al^3+^, Mn^2+^, Fe^3+^, Co^2+^, Ni^2+^, Cu^2+^, Zn^2+^, Ag^+^, Cd^2+^, Hg^2+^, and Pt^4+^). Remarkably, P**1a**/**2a** exhibited an exclusive and drastic fluorescence quenching only upon the addition of Pd^2+^ and Au^3+^, whereas the other competing ions induced negligible changes in the emission intensity (Figure 4A,B). This screening firmly establishes its exceptional selectivity and robust anti-interference capability towards noble metal ions. Subsequently, quantitative fluorescence titration experiments were conducted. Upon the incremental addition of Pd^2+^ and Au^3+^ within the concentration range of 0 to 20 μM and 0 to 36 μM, respectively, the photoluminescence intensity of P**1a**/**2a** underwent a progressive and continuous decrease (Figure 4C,E). A well-defined linear correlation between the relative emission intensity and the metal ion concentration was established in the low-concentration regime. Based on the standard 3σ/k and 10σ/k methods, the limits of detection (LOD) for Pd^2+^ and Au^3+^ were determined to be 7.11 × 10^−7^ M and 1.06 × 10^−6^ M, respectively (Figure 4D,F), while the corresponding limits of quantification (LOQ) were calculated to be 2.37 × 10^−6^ M and 3.53 × 10^−6^ M, respectively. These impressively low sub-micromolar and micromolar LOD values unambiguously demonstrate the probe’s exceptional sensitivity, rendering it highly capable of the real-time trace detection of noble metal ions [1,48]. We speculate that this exceptional selectivity stems from Pearson’s hard–soft acid–base (HSAB) theory. The poly(vinyl sulfide) backbone of P**1a**/**2a** is richly endowed with sulfur atoms (soft bases), which presumably possess a strong, specific binding affinity toward noble transition metal ions like Pd^2+^ and Au^3+^ (soft acids). Upon this likely coordination, the strong fluorescence of the TPE core is drastically quenched. We hypothesize that this quenching phenomenon is primarily driven by the heavy-atom effect (HAE) induced by the coordinated Pd and Au centers. Such an effect would facilitate rapid intersystem crossing (ISC) from the emissive excited singlet state to non-emissive triplet states. Furthermore, a photoinduced electron transfer (PET) process between the excited fluorophore and the unfilled d-orbitals of the transition metals might also be synergistically involved in the non-radiative decay [1,49].

**Figure 5:**
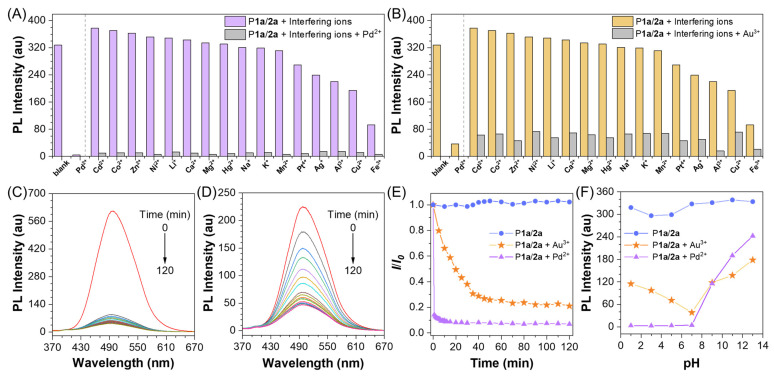
Competitive metal ion interference study for the target detection. PL intensity responses of the probe P**1a**/**2a** to various background metal ions at 500 nm followed by the subsequent addition of (**A**) Pd^2+^ and (**B**) Au^3+^. In both panels, the dark-colored bars (lavender purple in (**A**), apricot yellow in (**B**)) represent the initial PL intensity of P**1a**/**2a** in the presence of background ions alone. The adjacent light-gray bars indicate the quenched PL intensity after adding the respective target ions. The vertical dashed line separates the blank control from the complex interference groups. The drastic and consistent drop in PL intensity across all tested mixtures demonstrates the robust anti-interference capability of the probe. Time-dependent PL spectra of P**1a**/**2a** upon the addition of (**C**) Pd^2+^ and (**D**) Au^3+^ over 120 min. (**E**) Corresponding time-dependent relative PL intensity (*I*/*I*_0_) at 500 nm for the blank P**1a**/**2a**, P**1a**/**2a** + Pd^2+^, and P**1a**/**2a** + Au^3+^, where *I*_0_ and *I* are the PL intensities of the probe before and after adding the target metal ions, respectively. (**F**) Effect of pH on the PL intensity at 500 nm of P**1a**/**2a** in the absence and presence of Pd^2+^ and Au^3+^. Experimental conditions: [P**1a**/**2a**] = 40 μM in THF/H_2_O mixture (*v*/*v* = 2:8), [metal ions] = 100 μM, *λ*_ex_ = 350 nm.

To thoroughly evaluate the practical applicability of P**1a**/**2a** in complex environmental matrices, its anti-interference capability and sensing kinetics were rigorously investigated. Selectivity profiling against 16 representative background ions revealed that the inherent photoluminescence of P**1a**/**2a** remained largely unaffected, with the exception of minor baseline fluctuations induced by strongly paramagnetic ions like Cu^2+^ and Fe^3+^ (Figure 5A,B). Crucially, subsequent exposure to Pd^2+^ or Au^3+^ in these mixed solutions still triggered dramatic fluorescence quenching. As summarized in Table S1, the quenching efficiency (QE) for Pd^2+^ remained exceptionally robust (>93.6%) across all tested competitive environments. Similarly, a highly reliable QE profile (predominantly >75%) was maintained for Au^3+^, unambiguously demonstrating that P**1a**/**2a** possesses excellent anti-interference capabilities for exclusive noble metal detection. Furthermore, time-dependent PL measurements revealed distinct kinetic behaviors for the two analytes (Figure 5C–E). The addition of Pd^2+^ induced an ultrafast turn-off response, with the emission intensity plummeting to its minimum within 1–2 min, facilitating real-time monitoring. In contrast, the quenching process for Au^3+^ proceeded more gradually, reaching a steady state after approximately 60 min, which likely reflects the different coordination kinetics and binding affinities between the sulfur-rich backbone and the respective metal centers. Finally, the environmental robustness of the sensor was validated via pH titrations (Figure 5F). While the pristine polymer exhibited a highly stable baseline emission across a broad pH range of 1–13, the specific quenching by Pd^2+^ and Au^3+^ was optimized under acidic to neutral conditions (pH 1–7). The discernible decrease in sensing sensitivity observed in highly alkaline media (pH > 8) can be reasonably ascribed to the competitive formation of insoluble metal hydroxides, which effectively impedes the crucial sulfur–metal coordination events.

In summary, P**1a**/**2a** demonstrates outstanding sensing performance for noble metal ions, driven by its inherent aggregation-induced emission characteristics and specific sulfur–metal coordination. The material exhibits exceptional selectivity and sensitivity toward palladium and gold ions, achieving impressively low limits of detection of 7.11 × 10^−7^ M and 1.06 × 10^−6^ M, respectively, and LOQ values of 2.37 × 10^−6^ M and 3.53 × 10^−6^ M, respectively. Furthermore, the probe displays robust anti-interference capabilities against a wide panel of competing background ions, ultrafast response kinetics for palladium within one to two minutes, and excellent environmental stability across a broad acidic to neutral pH range. These remarkable features collectively highlight the immense potential of this material for the reliable and real-time trace detection of precious metals in complex environmental matrices.

## 4. Conclusions

In summary, we have successfully established a novel, 100% atom economy, transition-metal-free alkyne–thiol click polymerization. Catalyzed by *^t^*BuOLi at 80 °C, this highly efficient protocol affords a series of poly(vinyl sulfide)s with high molecular weights and excellent yields. Driven by their densely aromatic and sulfur-rich structural features, the resulting PVSs exhibit outstanding thermal stability alongside exceptional light refractivity. Furthermore, by leveraging the abundant sulfur coordination sites and the inherent AIE characteristics, we successfully deployed the PVS aggregates as highly reliable fluorescent chemosensors. These sensors display remarkable anti-interference capabilities and environmental robustness for the exclusive detection of noble metal ions, achieving trace detection of Pd^2+^ and Au^3+^ with impressive limits of detection. Ultimately, this work not only provides a robust and sustainable synthetic methodology for constructing heteroatom-rich macromolecules but also underscores their promising potential in next-generation optical engineering and environmental monitoring.

## Figures and Tables

**Table 1:** Polymerization Results of terminal aryldiyne **1a** and benzyl dithiol **2a** ^a^.

Entry	Solvent	Solvent Ratio	*T* (°C)	Time (h)	Yield (%)	*M*_w_ ^b^ (g/mol)	PDI ^b^
Screening of different solvents							
1	EtOH/DMSO	1:1	60	12	71	8600	1.41
2	H_2_O/DMSO	1:1	60	12	trace	/	/
3	EtOH/DMF	1:1	60	12	52	6700	1.56
4	EtOH/DCM	1:1	60	12	20	7900	1.68
5	EtOH/DCE	1:1	60	12	trace	/	/
Screening of solvents volume ratios							
6	EtOH/DMSO	3:1	60	12	42	5600	1.36
7	EtOH/DMSO	2:1	60	12	58	7900	1.53
8	EtOH/DMSO	1:2	60	12	43	7700	1.92
9	EtOH/DMSO	1:3	60	12	44	4800	1.36
Screening of temperature							
10	EtOH/DMSO	1:1	50	12	41	5500	1.39
11	EtOH/DMSO	1:1	70	12	83	10,100	1.66
12	EtOH/DMSO	1:1	80	12	91	11,800	1.87
Screening of polymerization time							
13	EtOH/DMSO	1:1	80	6	58	6600	2.01
14	EtOH/DMSO	1:1	80	8	64	7900	1.52
15	EtOH/DMSO	1:1	80	10	76	8700	1.79

^a^ Carried out in air in the presence of *^t^*BuOLi. [**1a**] = [**2a**] = [*^t^*BuOLi] = 0.10 M. ^b^ Determined by GPC in THF on the basis of a linear polystyrene calibration. PDI = *M*_w_/*M*_n_.

## Data Availability

The original contributions presented in this study are included in the article. Further inquiries can be directed to the corresponding author.

## Supplementary Materials

The following supporting information can be downloaded at: https://www.mdpi.com/article/10.3390/polym18101202/s1, Figure S1. ^13^C NMR spectra of (A) **1a**, (B) **2a**, (C) model compound **4** and (D) P**1a**/**2a** in CDCl_3_. The solvent peaks were marked with asterisks; Figure S2. IR spectra of (A) P**1a**/**2a**, (B) P**1b**/**2a**, (C) P**1c**/**2a**, (D) P**1d**/**2a**, (E) P**1a**/**2b**; Figure S3. ^1^H NMR spectrum of P**1b**/**2a** in CDCl_3_. The solvent peaks were marked with asterisks; Figure S4. High-resolution mass spectrum (ESI-HRMS) of model compound **4**; Figure S5. DSC thermograms of P**1**/**2** recorded under Argon during the second heating cycle at a heating rate of 10 °C/min; Figure S6. (A) Absorption spectra of P**1**/**2** in THF solutions. (B) PL spectra of P**1a**/**2a**, P**1b**/**2a** and P**1a**/**2b** in THF solutions. Experimental conditions: [P**1**/**2**] = 40 μM, *λ*_ex_ = 350 nm; Figure S7. (A) PL spectra of P**1b**/**2a** in THF/H_2_O mixtures with different water fractions (*f*_w_, vol%). (B) Plot of the relative PL intensity (*I*/*I*_0_) at 500 nm versus the water fraction. *I*_0_ represents the PL intensity in pure THF. Experimental conditions: [P**1b**/**2a**] = 40 μM, *λ*_ex_ = 350 nm; Table S1. Fluorescence quenching efficiencies (QE) of the probe toward Pd^2+^ and Au^3+^ in the presence of various background metal ions. Figures S8–S17. ^1^H NMR and ^13^C NMR spectra of monomer **1a**–**1d** and compound **4** in CDCl_3_.
