# Supplementary material for: Transition-Metal-Free Click Polymerization Toward Poly(vinyl sulfide)s Endowed with AIE-Driven Noble Metal Sensing
Source: Polymers (Basel). 2026 May 14;18(10):1202. doi: 10.3390/polym18101202 (PMC13210572; doi:10.3390/polym18101202)
Supplement: Supplementary file 1 [file polymers-18-01202-s001.zip › polymers-4284938-supplementary.pdf]

Supporting Information

Transition-Metal-Free Click Polymerization Toward Poly(vinyl sulfide)s Endowed with AIE-Driven Noble Metal Sensing

Liangcong Fan, Peisen Xu, Hongyu Wang, Zhifeng Cai, Juan Zuo, Cong Liu, Xiaohang Tan, Fengxiong Long, Hao Luo,* and Qingqing Gao,*

School of Materials Science and Engineering, Xiamen University of Technology, Xiamen 361024, China

fanliangcong@gmail.com (L. F.); 15939446407@139.com (P. X.); whongyu605@gmail.com (H. W.); czf3430324293@163.com (Z. C.); zuojuan@xmut.edu.cn (J. Z.); cuigu808@126.com (C. L.); 15959445032@163.com (X. T.); 15318720649@163.com (F. L.); luohao@xmut.edu.cn (H. L.); qgao@connect.ust.hk (Q. G)

* Correspondence: Correspondence: luohao@xmut.edu.cn (H.L.); qgao@connect.ust.hk

Table of Contents

Figure S1. ^{13}C NMR spectra of (A) **1a**, (B) **2a**, (C) model compound **4** and (D) P**1a/2a** in $CDCl_3$. The solvent peaks were marked with asterisks. S3
Figure S2. IR spectra of (A) P**1a/2a**, (B) P**1b/2a**, (C) P**1c/2a**, (D) P**1d/2a**, (E) P**1a/2b** S4
Figure S3. ^{1}H NMR spectrum of P**1b/2a** in $CDCl_3$. The solvent peaks were marked with asterisks...S4
Figure S4. High-resolution mass spectrum (ESI-HRMS) of model compound **4**.......S5
Figure S5. DSC thermograms of P**1/2** recorded under Argon during the second heating cycle at a heating rate of 10 °C/min..S5
Figure S6. (A) Absorption spectra of P**1/2** in THF solutions. (B) PL spectra of P**1a/2a,** P**1b/2a** and P**1a/2b** in THF solutions. Experimental conditions: [P**1/2**] = 40 μM, λ_{ex} = 350 nm. S6
Figure S7. (A) PL spectra of P**1b/2a** in THF/H_2O mixtures with different water fractions (f_w, vol%). (B) Plot of the relative PL intensity (I/I_0) at 500 nm versus the water fraction. I_0 represents the PL intensity in pure THF. Experimental conditions: [P**1b/2a**] = 40 μM, λ_{ex} = 350 nm. S6
Table S1. Fluorescence quenching efficiencies (QE) of the probe toward Pd^{2+} and Au^{3+} in the presence of various background metal ions. S7
Figure S8. ^{1}H NMR spectrum of monomer **1a** in $CDCl_3$. S8
Figure S9. ^{13}C NMR spectrum of monomer **1a** in $CDCl_3$. S8
Figure S10. ^{1}H NMR spectrum of monomer **1b** in $CDCl_3$. S9
Figure S11. ^{13}C NMR spectrum of monomer **1b** in $CDCl_3$. S9
Figure S12. ^{1}H NMR spectrum of monomer **1c** in $CDCl_3$. S10
Figure S13. ^{13}C NMR spectrum of monomer **1c** in $CDCl_3$. S10
Figure S14. ^{1}H NMR spectrum of monomer **1d** in $CDCl_3$. S11
Figure S15. ^{13}C NMR spectrum of monomer **1d** in $CDCl_3$. S11
Figure S16. ^{1}H NMR spectrum of compound **4** in $CDCl_3$. S12
Figure S17. ^{13}C NMR spectrum of compound **4** in $CDCl_3$. S12

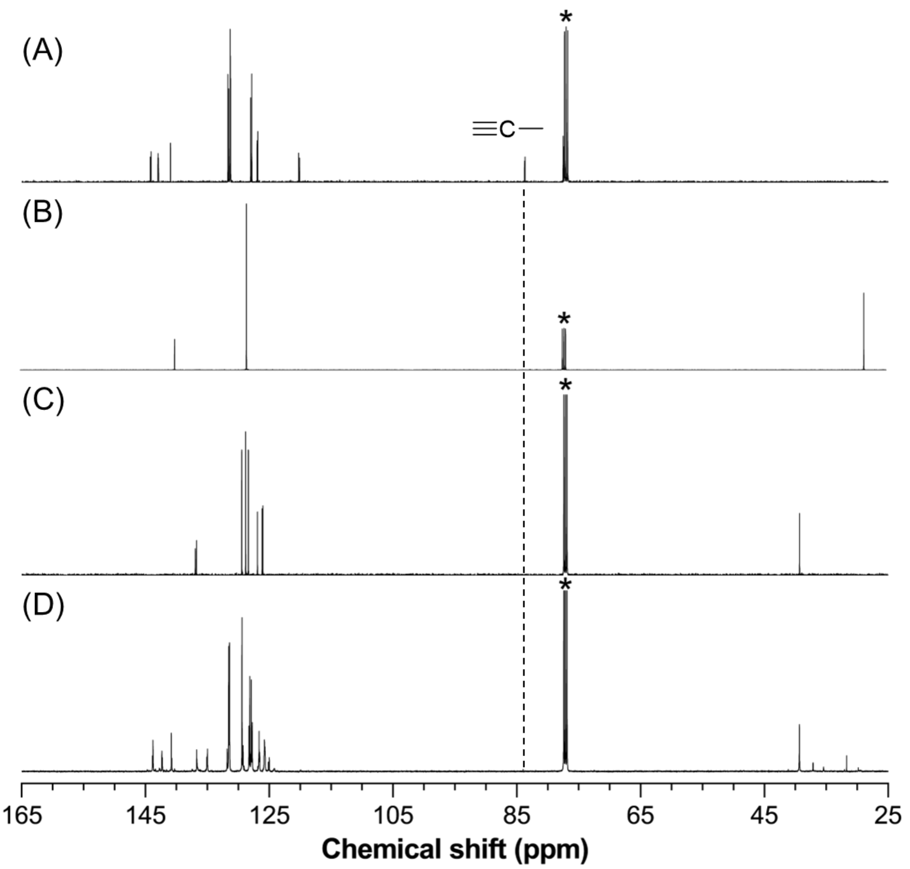

Figure S1. ^{13}C NMR spectra of (A) **1a**, (B) **2a**, (C) model compound **4** and (D) P**1a/2a** in $CDCl_3$. The solvent peaks were marked with asterisks.

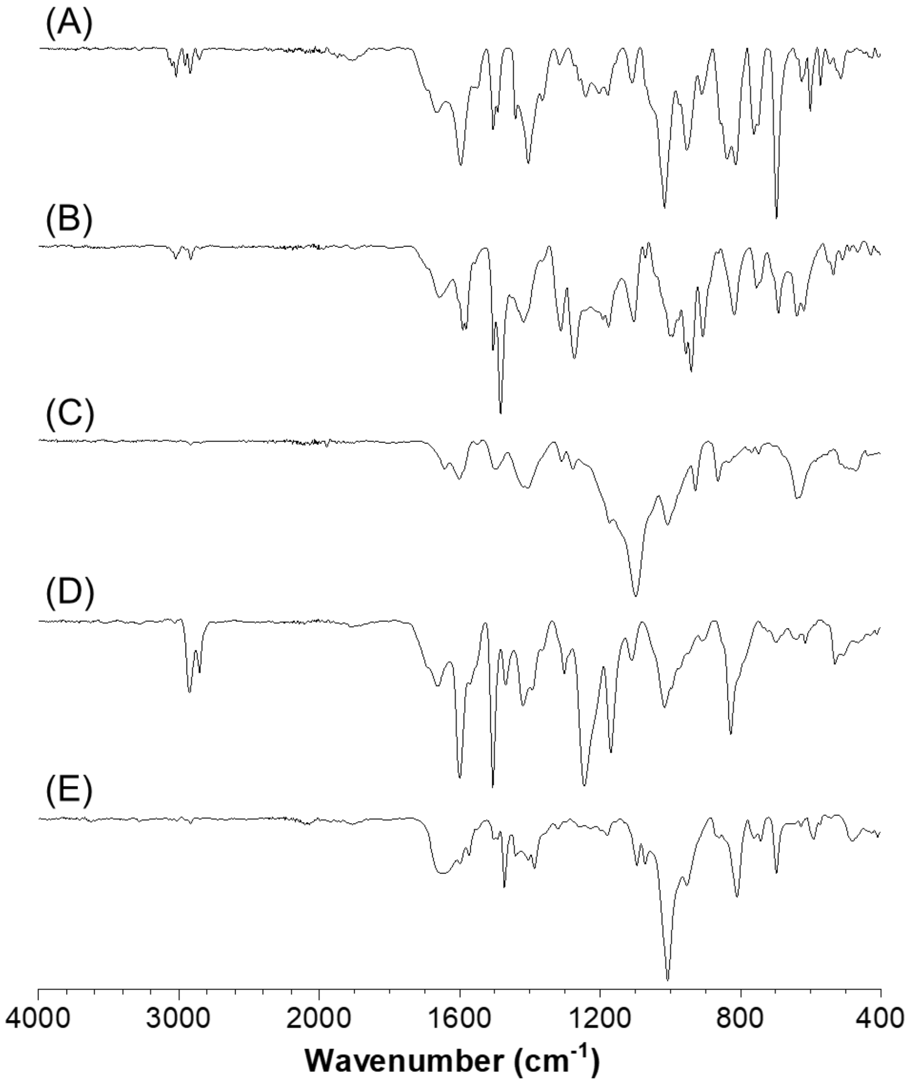

Figure S2. IR spectra of (A) P**1a**/**2a**, (B) P**1b**/**2a**, (C) P**1c**/**2a**, (D) P**1d**/**2a**, (E) P**1a**/**2b**

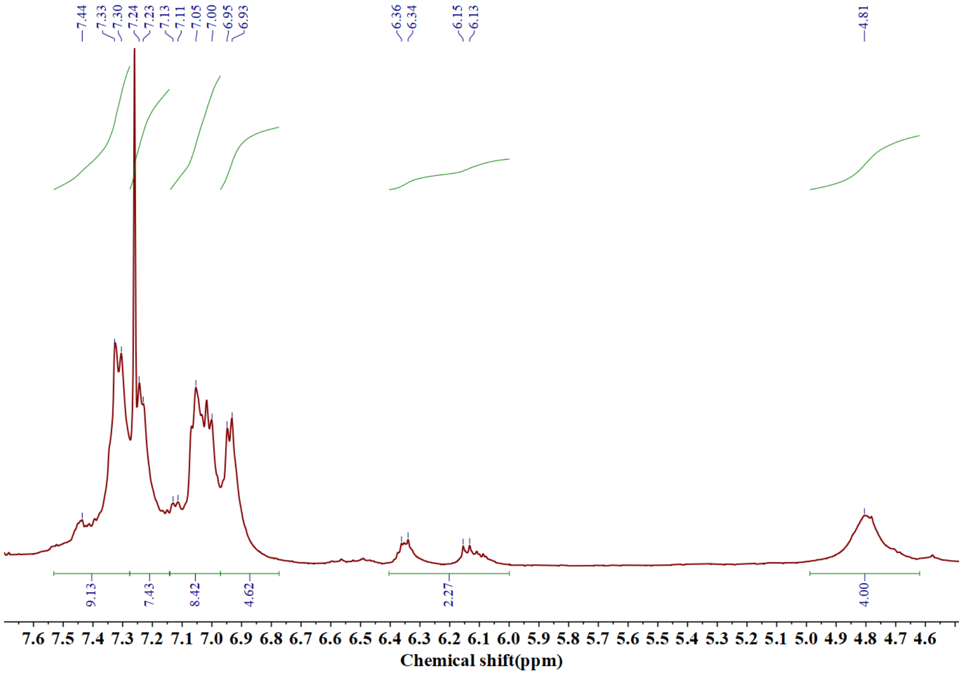

Figure S3. ^{1}H NMR spectrum of P**1b**/**2a** in $CDCl_3$. The solvent peaks were marked

with asterisks.

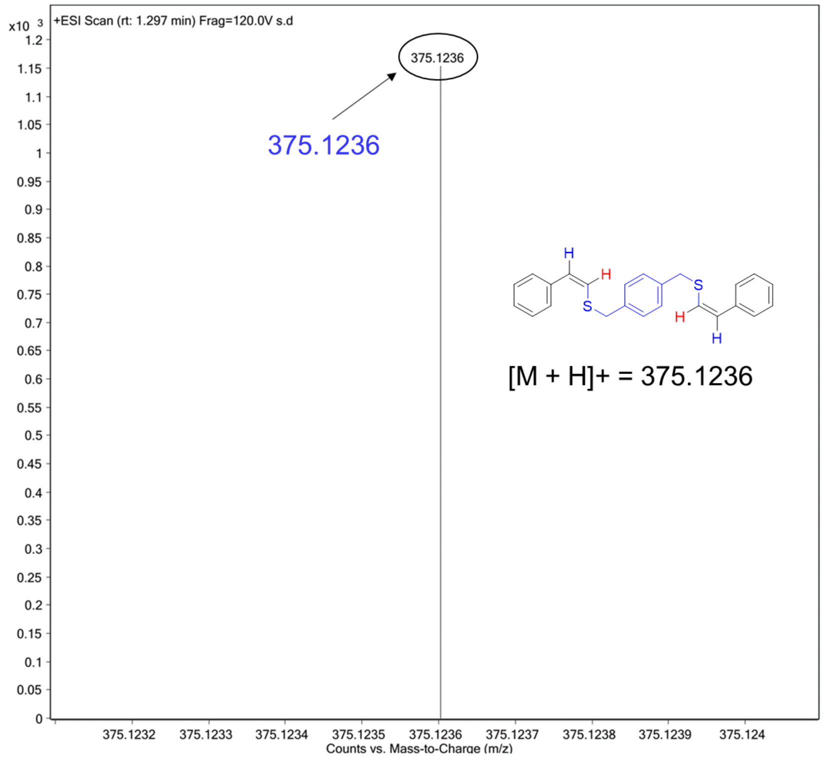

Figure S4. High-resolution mass spectrum (ESI-HRMS) of model compound **4**.

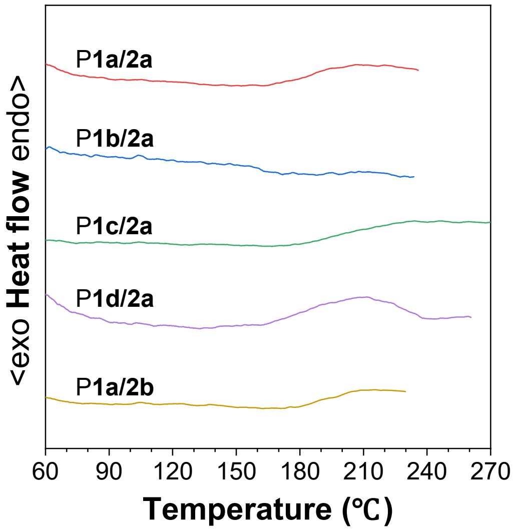

Figure S5. DSC thermograms of P**1**/**2** recorded under Argon during the second heating cycle at a heating rate of 10 °C/min.

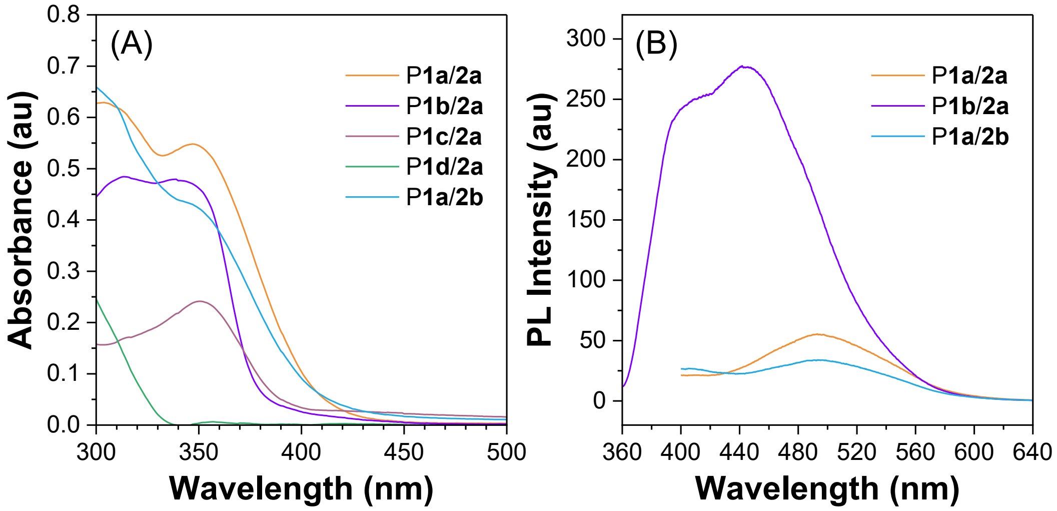

Figure S6. (A) Absorption spectra of P**1**/**2** in THF solutions. (B) PL spectra of P**1a**/**2a**, P**1b**/**2a** and P**1a**/**2b** in THF solutions. Experimental conditions: [P**1**/**2**] = 40 μM, λ_{ex} = 350 nm.

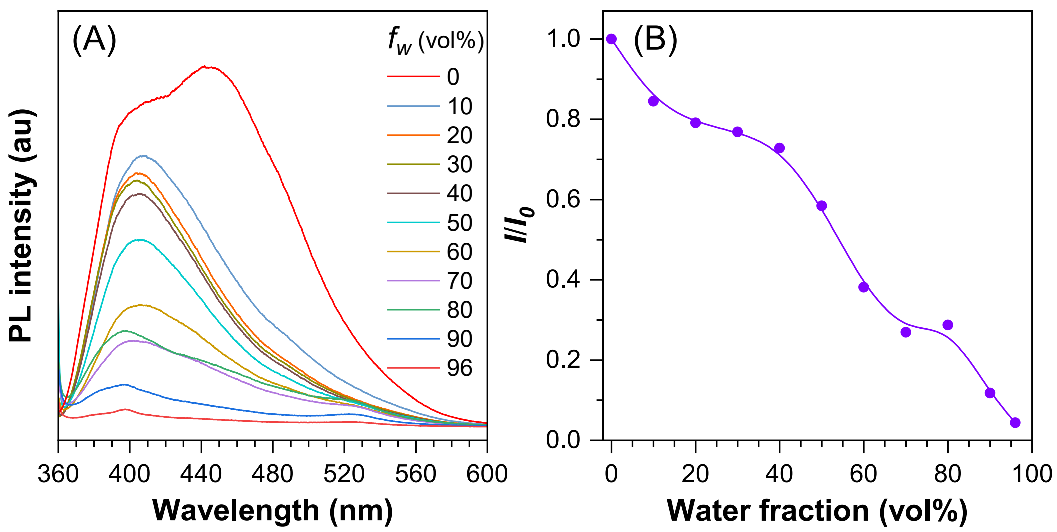

Figure S7. (A) PL spectra of P**1b**/**2a** in THF/H_2O mixtures with different water fractions (f_w, vol%). (B) Plot of the relative PL intensity (I/I_0) at 500 nm versus the water fraction. I_0 represents the PL intensity in pure THF. Experimental conditions: [P**1b**/**2a**] = 40 μM, λ_{ex} = 350 nm.

Table S1. Fluorescence quenching efficiencies (QE) of the probe toward Pd^{2+} and Au^{3+} in the presence of various background metal ions.

Background Ions	For Pd^{2+}			For Au^{3+}		
	I_0^a	I^b	QE[c] (%)	I_0^a	I^b	QE[c] (%)
blank	327.7	4.4	98.7	327.7	36.75	88.8
Cd^{2+}	377.9	9.9	97.4	377.9	62.8	83.4
Co^{2+}	370.7	10.8	97.1	370.7	66.3	82.1
Zn^{2+}	363.3	10.8	97.0	363.3	46.6	87.2
Ni^{2+}	351.9	6.1	98.3	351.9	73.5	79.1
Li^{+}	348.6	13.0	96.3	348.6	55.2	84.2
Ca^{2+}	343.4	9.8	97.2	343.4	69.2	79.9
Mg^{2+}	334.1	6.0	98.2	334.1	63.8	80.9
Hg^{2+}	331.0	8.1	97.6	331.0	54.8	63.5
Na^{+}	321.2	10.5	96.7	321.2	65.8	79.5
K+	319.6	11.7	96.3	319.6	67.3	78.9
Mn^{2+}	311.6	6.1	98.0	311.6	67.2	78.4
Pt^{4+}	269.6	8.1	97.0	269.6	46.6	82.7
Ag^{+}	239.6	14.2	94.1	239.6	50.4	79.0
Al^{3+}	220.6	14.2	93.6	220.6	15.8	92.9
Cu^{2+}	194.6	11.0	94.4	194.6	71.2	63.4
Fe^{3+}	92.6	5.5	94.1	92.6	21.2	77.1

[a] I_0 represents the initial PL intensity of the probe in the presence of background metal ions (100 μM). [b] I represents the PL intensity after the subsequent addition of the target ion (Pd^{2+} or Au^{3+}, 100 μM). [c] The quenching efficiency (QE) was calculated according to the equation: $QE = [(I_0 - I)/I_0] \times 100\%$. Experimental conditions: [P**1a**/**2a**] = 40 μM, measured in THF/H_2O mixture (v/v = 2:8) at room temperature, λ_{ex} = 350 nm.

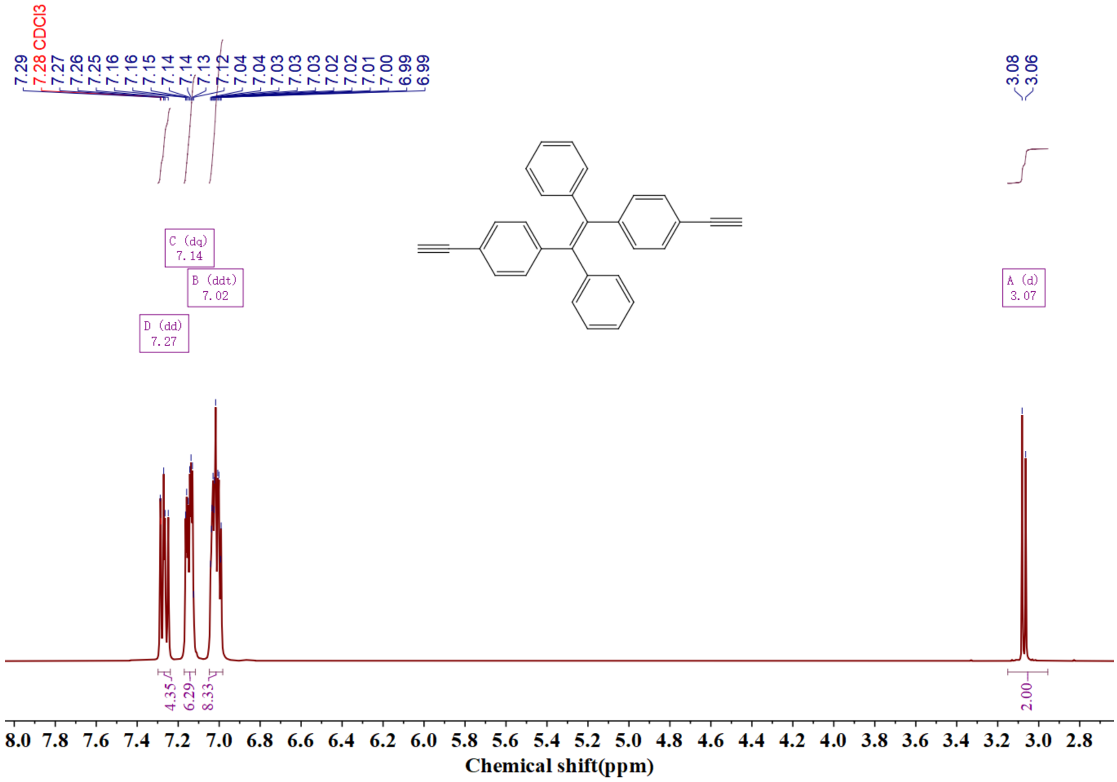

Figure S8. ^{1}H NMR spectrum of monomer **1a** in $CDCl_3$.

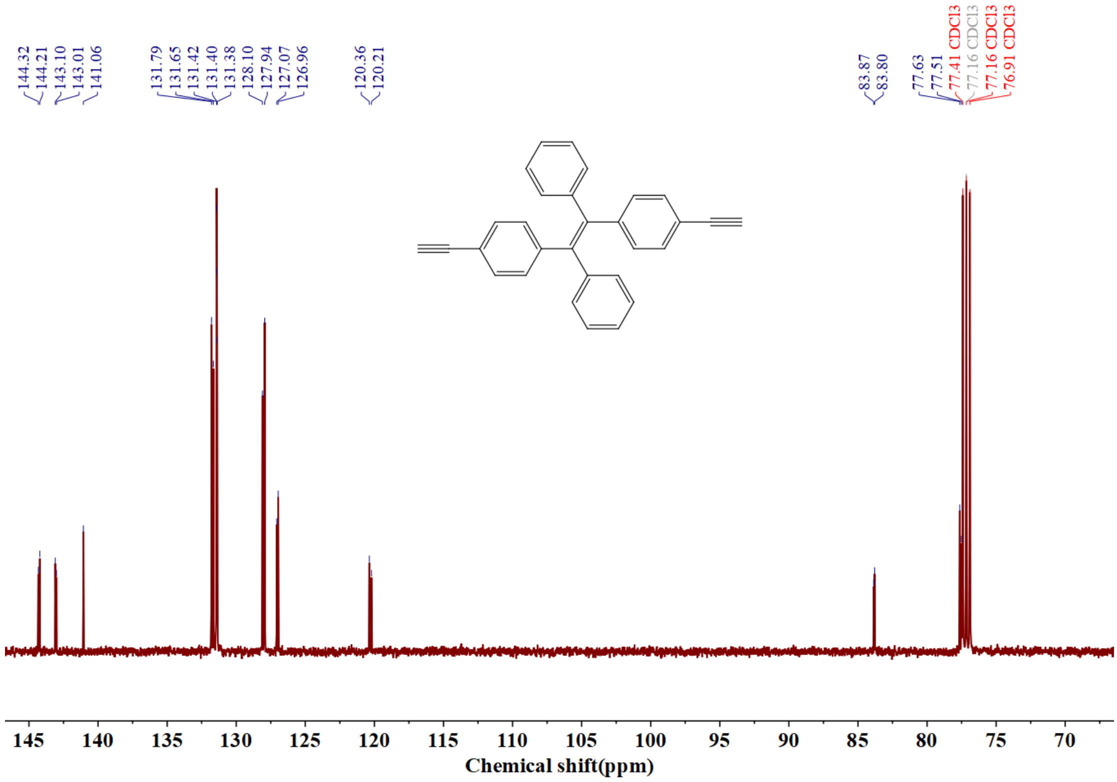

Figure S9. ^{13}C NMR spectrum of monomer **1a** in $CDCl_3$.

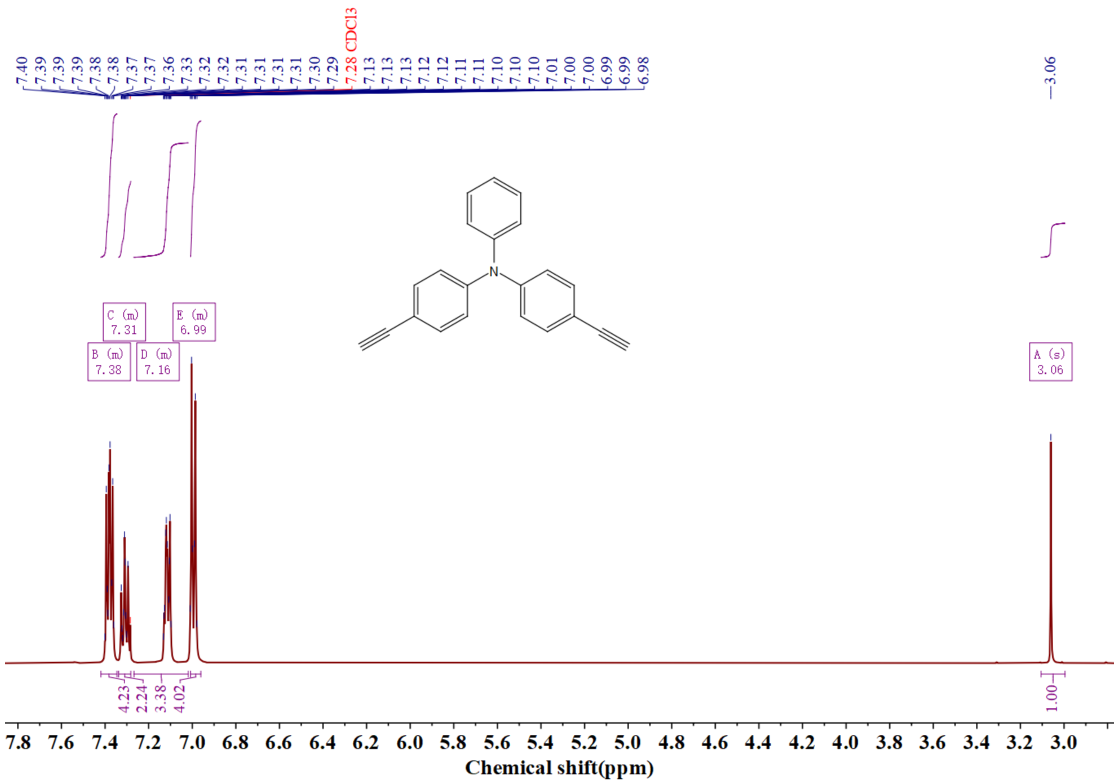

Figure S10. ^{1}H NMR spectrum of monomer **1b** in $CDCl_3$.

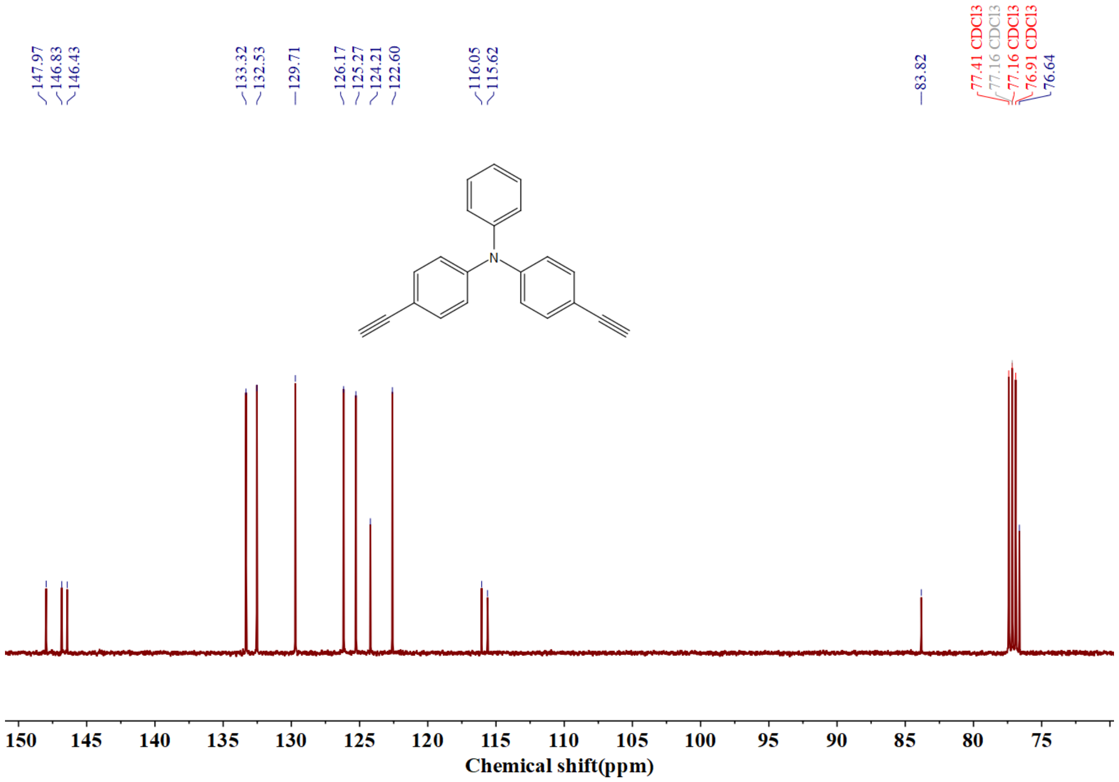

Figure S11. ^{13}C NMR spectrum of monomer **1b** in $CDCl_3$.

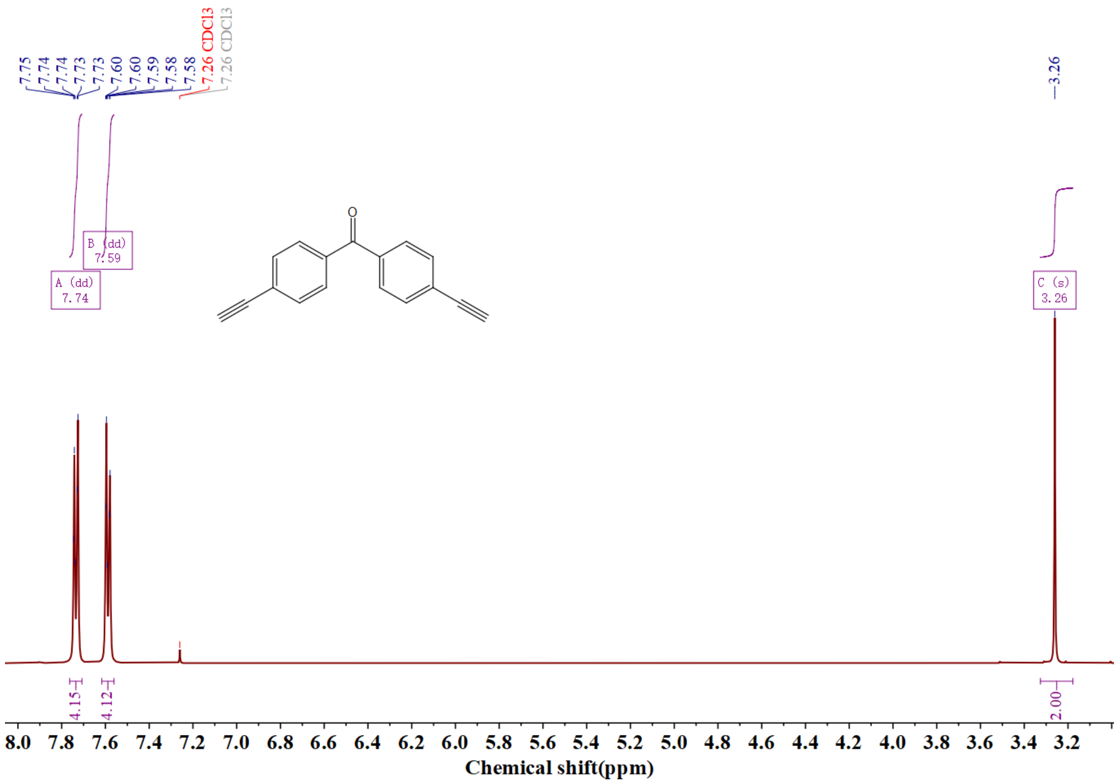

Figure S12. ^{1}H NMR spectrum of monomer **1c** in $CDCl_3$.

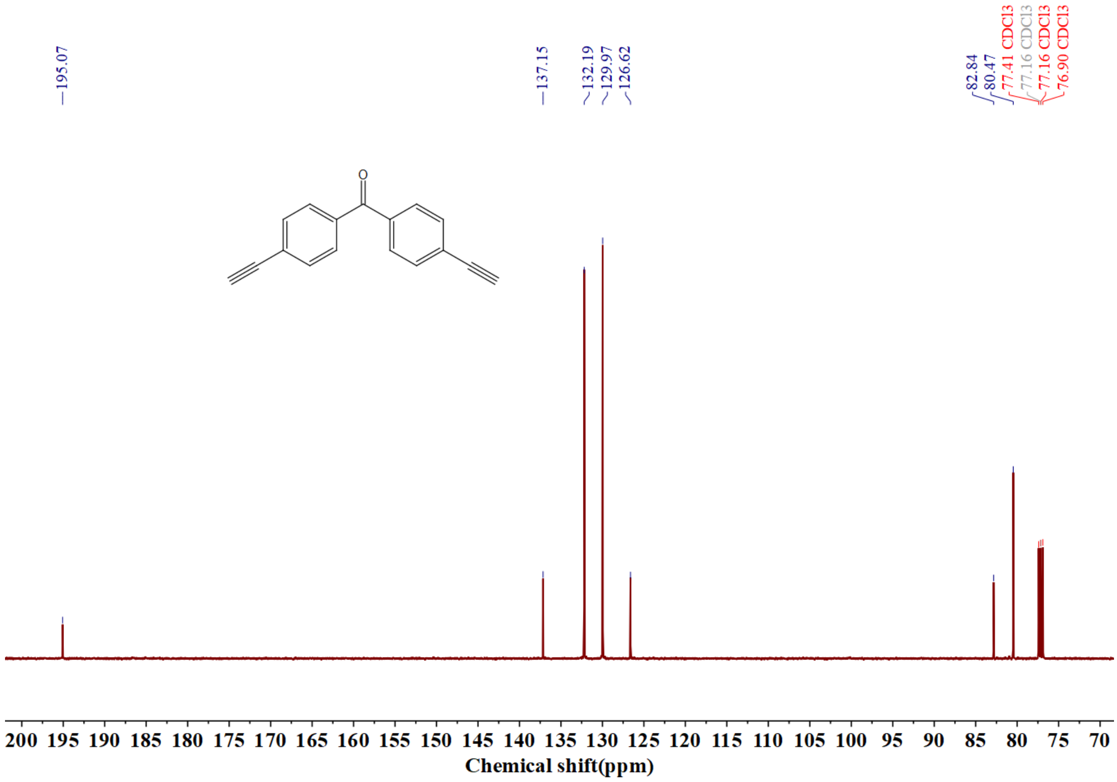

Figure S13. ^{13}C NMR spectrum of monomer **1c** in $CDCl_3$.

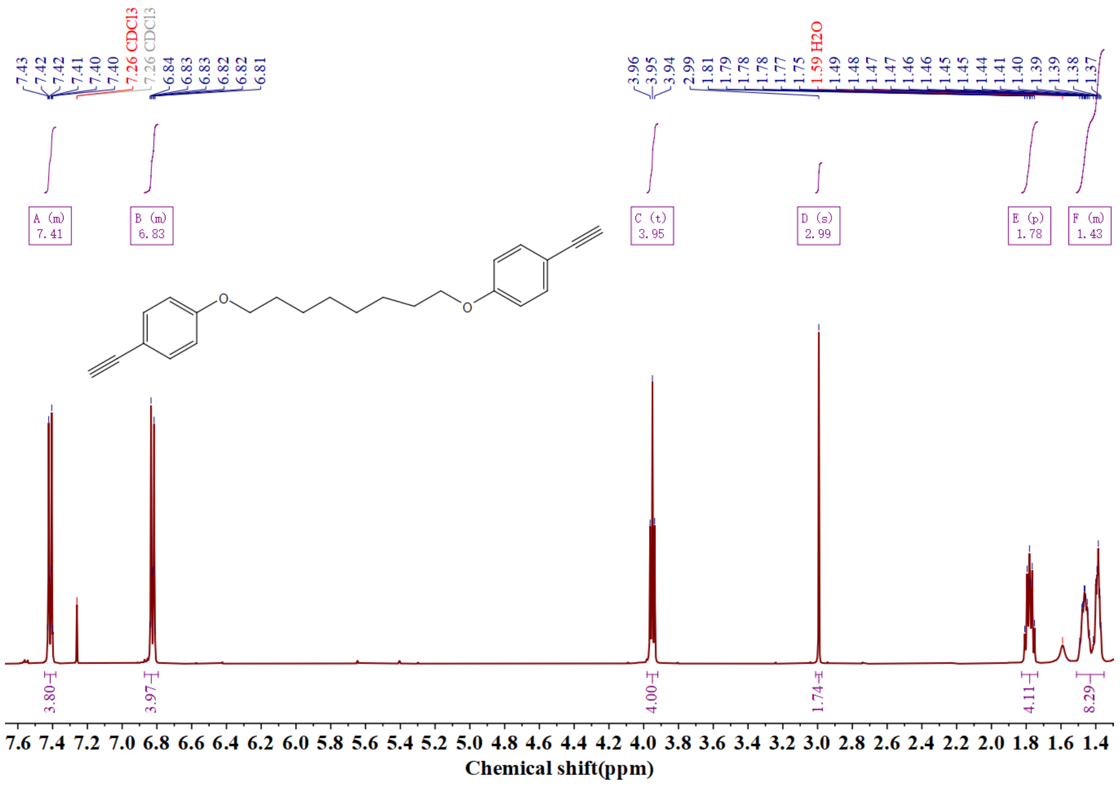

Figure S14. ^{1}H NMR spectrum of monomer **1d** in $CDCl_3$.

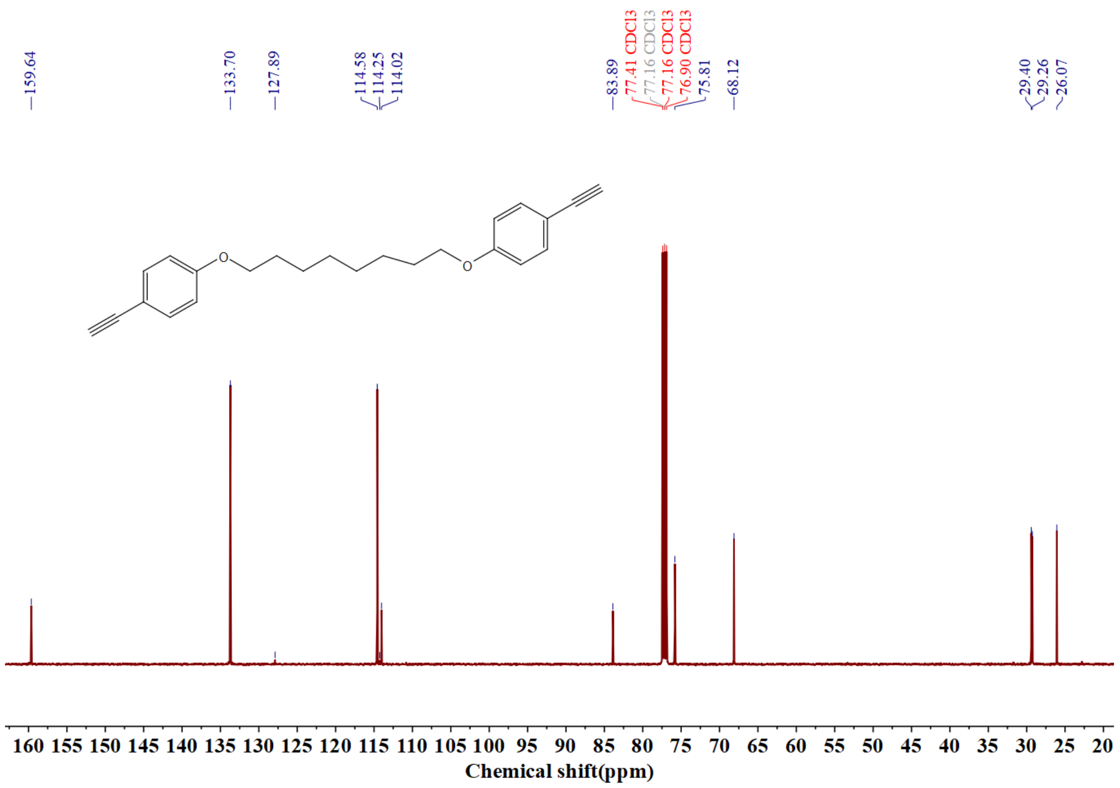

Figure S15. ^{13}C NMR spectrum of monomer **1d** in $CDCl_3$.

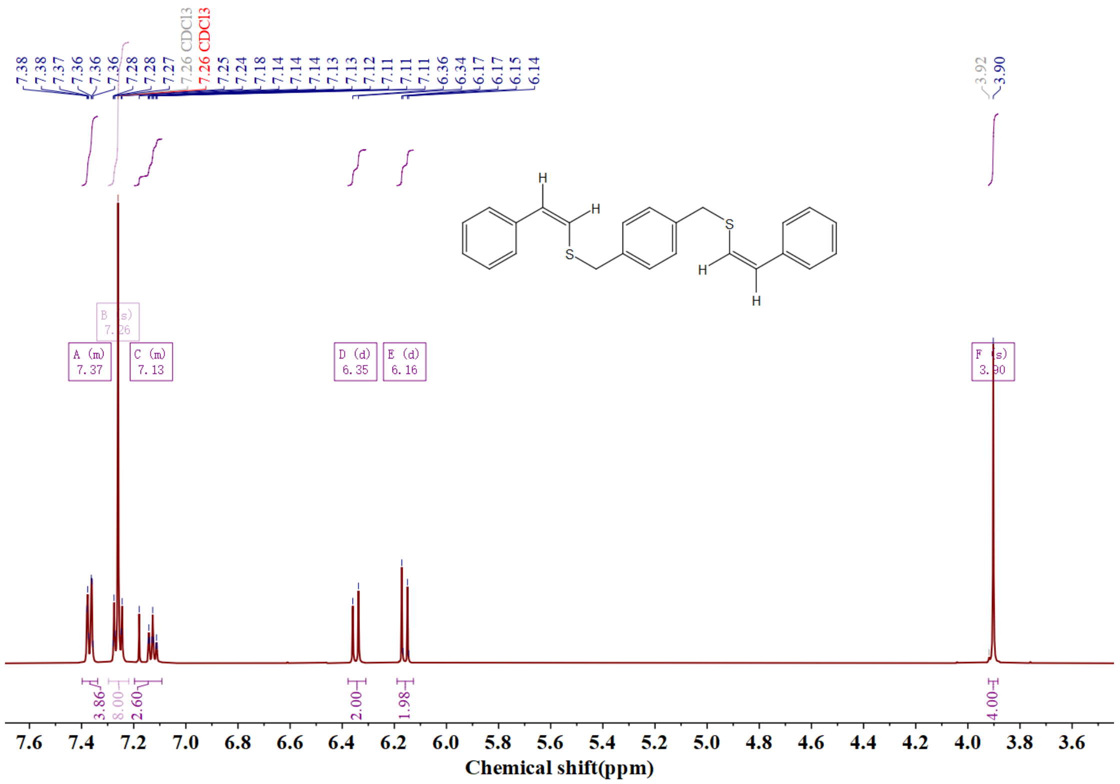

Figure S16. ^{1}H NMR spectrum of compound **4** in $CDCl_3$.

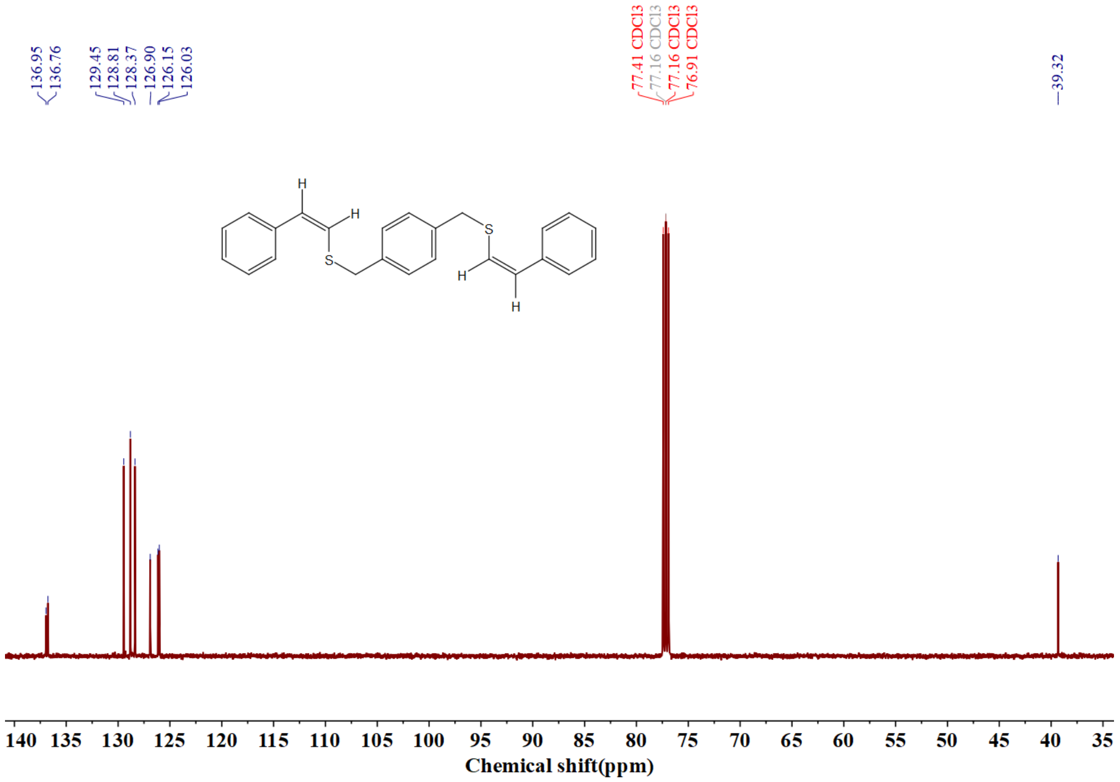

Figure S17. ^{13}C NMR spectrum of compound **4** in $CDCl_3$.
